# Advances on MXene-Based Memristors for Neuromorphic Computing: A Review on Synthesis, Mechanisms, and Future Directions

**DOI:** 10.1021/acsnano.4c03264

**Published:** 2024-08-07

**Authors:** Henrique Teixeira, Catarina Dias, Andreia Vieira Silva, João Ventura

**Affiliations:** IFIMUP, Departamento de Física e Astronomia, Faculdade de Ciências, Universidade do Porto, Rua do Campo Alegre s/n, 4169-007, Porto, Portugal

**Keywords:** Artificial Synapse, Memristor, MXene, Neuromorphic Computing, Resistive Switching, 2D Materials, van der Waals, Memory, Neural Networks

## Abstract

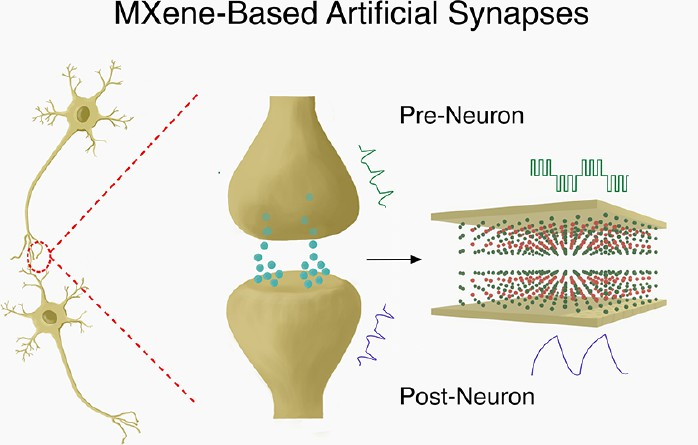

Neuromorphic computing seeks to replicate the capabilities of parallel processing, progressive learning, and inference while retaining low power consumption by drawing inspiration from the human brain. By further overcoming the constraints imposed by the traditional von Neumann architecture, this innovative approach has the potential to revolutionize modern computing systems. Memristors have emerged as a solution to implement neuromorphic computing in hardware, with research based on developing functional materials for resistive switching performance enhancement. Recently, two-dimensional MXenes, a family of transition metal carbides, nitrides, and carbonitrides, have begun to be integrated into these devices to achieve synaptic emulation. MXene-based memristors have already demonstrated diverse neuromorphic characteristics while enhancing the stability and reducing power consumption. The possibility of changing the physicochemical properties through modifications of the surface terminations, bandgap, interlayer spacing, and oxidation for each existing MXene makes them very promising. Here, recent advancements in MXene synthesis, device fabrication, and characterization of MXene-based neuromorphic artificial synapses are discussed. Then, we focus on understanding the resistive switching mechanisms and how they connect with theoretical and experimental data, along with the innovations made during the fabrication process. Additionally, we provide an in-depth review of the neuromorphic performance, making a connection with the resistive switching mechanism, along with a compendium of each relevant performance factor for nonvolatile and volatile applications. Finally, we state the remaining challenges in MXene-based devices for artificial synapses and the next steps that could be taken for future development.

## Introduction

**Figure 1:**
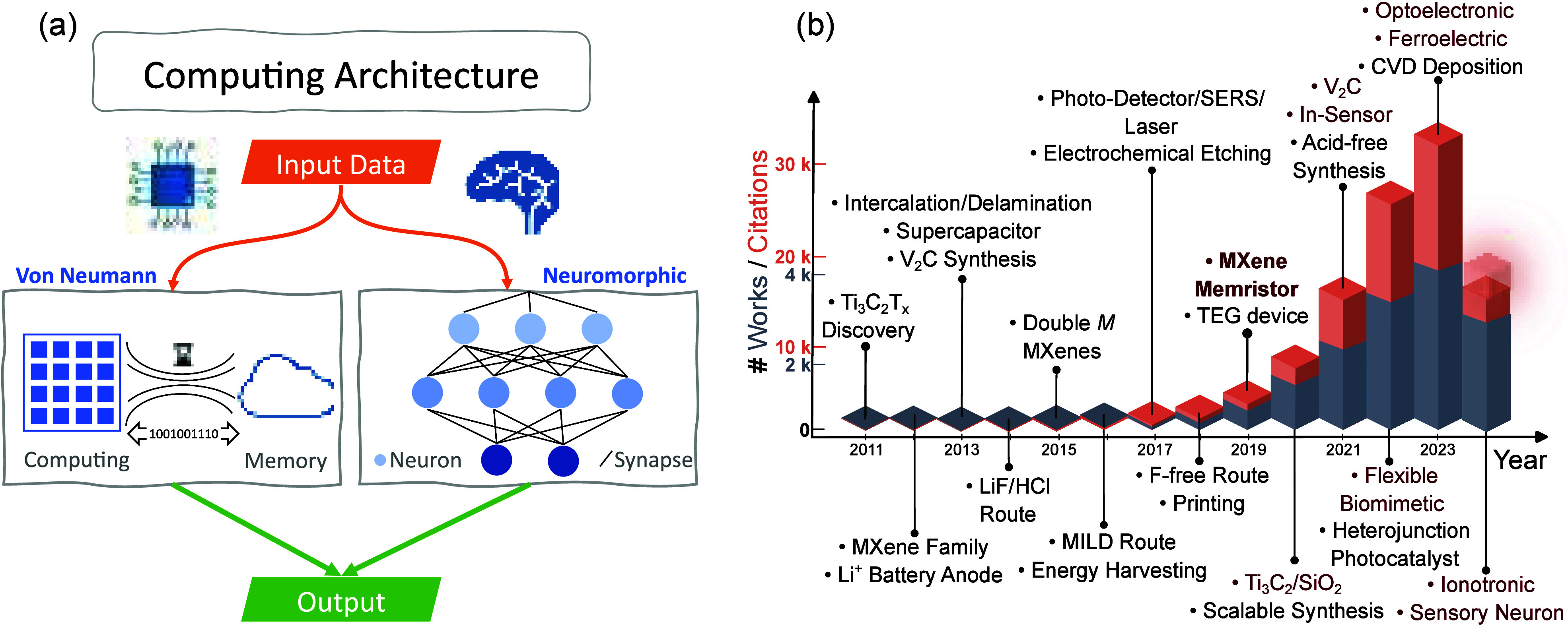
(a) Comparison between traditional (digital) von Neumann and (analog) neuromorphic computing architectures. (b) Main achievements in MXene research since discovery, with all MXene-based memristors in red, depicting the number of citations (number of works and respective citations obtained from Scopus,^4^ taken in June 2024).

In this era of “Big Data”, there is a growing interest in exploring solutions based on artificial intelligence (AI) such as artificial neural networks (ANNs). However, these AI-software approaches are deployed in computing units based on the von Neumann architecture, which is reaching its inherent performance bottleneck,^1^ due to data storage and computing units being physically separated. On the other hand, the human brain is capable of memory storage and learning in the same substrate, using its vast network of neurons and synapses to avoid data migration. The schematics in Figure 1(a) compares the two computing architectures. Furthermore, the human brain only requires approximately 20 W (0.3 kWh per day) to operate, against the ever-increasing energy hungry AI applications such as ChatGPT (260 KWh per day). Therefore, using neuromorphic computing architectures that seek to emulate the brain at the hardware level by mimicking the structure and function of biological neural networks in artificial computing systems or analog circuitry is a highly attractive solution.^2,3^

One of the key components in neuromorphic computing is the dynamic weights of synapses that connect neurons and allow data to be classified, emulating the strength of biological synapses. The large-scale assembly of such adaptive switches into electronic systems is rapidly evolving due to the recent discovery of the memristor.^5^ A memristor is defined as a two-terminal device where resistive switching (RS) allows dynamic conductance states to occur. This effect was already shown to mimic advanced biological learning rules such as short/long-term synaptic plasticity, Hebbian learning, and dendritic integration, among others.^1,6−10^

The first memristive devices were based on metal–insulator–metal stacks that still display low reproducibility between devices and temporal variations due to the intrinsic stochastic switching mechanism of the insulating materials used.^1,3,11^ The integration of low-dimensional materials in memristors was shown to enhance memory and neuromorphic properties such as higher switching control, higher spatial, and temporal reproducibility but also lower power consumption and fabrication cost.^7,12−14^ Besides, the integration of two-dimensional (2D) materials (e.g., MoS_2_, WSe_2_, WS_2_, graphene) in memristors^15^ provides easy device scaling, due to their atomic-scale thickness and the ability to form van der Waals heterostructures.^7,16,17^

MXenes are a family of 2D materials discovered in 2011,^18^ composed by transition metal carbonitrides, carbides, and nitrides obtained through etching the A layer from the MAX phase.^19^ The name MXenes emphasizes the morphological similarity to graphene and that this type of material is prepared from a MAX phase precursor. They have found use in a vast range of applications such as water purification,^20^ electromagnetic interference shielding,^21^ transparent and flexible electrodes,^22^ high-performance supercapacitors, biosensors^23,24^ (e.g., SARS-COV-2 detector,^25^ sweat-based sensors^26^), and storage devices.^27−29^ A timeline of the MXene research is shown in Figure 1(b). Since 2019, the integration of MXenes in these neuromorphic devices has gained significant interest, especially due to the obtained performance enhancements, along with their excellent charge trapping capability and electrical conductivity.^11,15^ In comparison with 3D materials for the switching layer, MXenes benefit from the reduced dimension, which offers better device integration and further decreases operation voltages and power consumption. Furthermore, as will be seen in detail below, the introduction of MXenes in memristors^30−32^ also leads to improved device performance. When compared to other 2D materials, such as transition metal dichalcogenides (TMDs), MXenes present unique chemical and physical properties such as hydrophilic surfaces that enable high chemical stability,^33,34^ metallic conduction, better optical transport properties due to the higher density of states at the Fermi level,^35,36^ and tunable surface terminations, which enable one to tailor properties such as work function or surface electronegativity.^34,36−39^ Moreover, during the etching and assembly stage, a large number of variables can influence the MXene properties, namely, surface terminations, interlayer spacing, flake size, or defects, meaning that even the same MXene material can have different chemical and physical properties,^40−43^ while at the same time there is an extremely large material family to explore. All of these considerations, coupled with versatile and inexpensive fabrication methods (spin/spray/dip-coating, printing)^29,44−46^ that prevent unwanted defects and damage typical of high-energy deposition methods,^47^ place MXenes as extremely promising materials for neuromorphic computing in-hardware.

This review begins with the fabrication methods of MXene-based memristors, analyzing the different etching and deposition methods, as well as variations in these processes. Then, the characterization techniques more appropriate for each stage of these processes are detailed. The influence of the fabrication process on the switching mechanism is analyzed together with the impact of the different tuning processes during the fabrication stage on these mechanisms. Finally, the most promising applications of the vast MXene family in neuromorphic applications are discussed, in particular their performance as artificial synapses, either in volatile or nonvolatile regimes.

## Biological Learning Mechanisms

**Figure 2:**
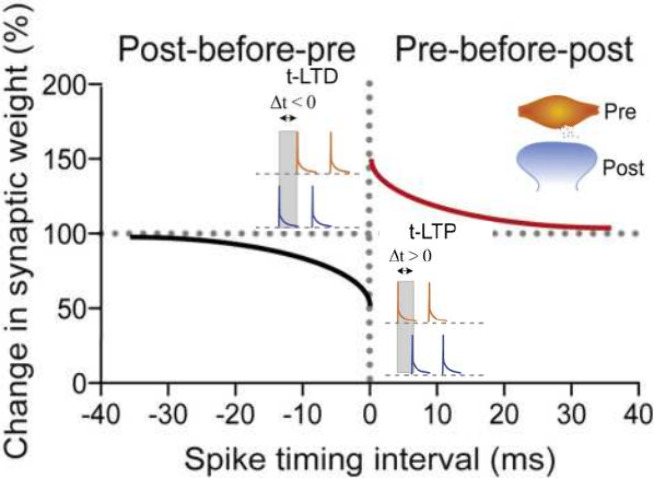
Change in long-term synaptic strength regarding timing between pre- and post-neuron firing. Reprinted in part with permission from ref (52). Copyright 2019 Elsevier.

The brain is the most complex human organ, capable of processing and memorizing information at extremely low power. It consists of a network of two fundamental components: neurons and synapses. Neurons are excitable nerve cells that communicate with each other through the synapses. Neuron excitation (action potential) occurs if the total cumulative charge arriving from the different synaptic connections reaches a certain threshold. Synaptic strength (or weight) then relates to the amount of voltage (or current) that an action potential of a presynaptic neuron produces in the postsynaptic neuron. It is therefore responsible for the bonding of two neurons (in a manner that can enhance or suppress signal transmission) and for the storage of biological memory.^48^ This strength can be changed and modulated through neural activity in a process called synaptic plasticity, which is the basis of learning.^49,50^ Several models have been developed to understand the workings of the brain, with a special emphasis on its learning mechanisms. In 1949, D. Hebb postulated that “neurons that fire together, wire together”, meaning that, if a neuron consistently fires to a subsequent one, their connection will strengthen.^51^ Later, the spike-time-dependent plasticity (STDP) model introduced firing temporal order as another factor that impacts learning. It states that the timing in which firing occurs impacts the amplitude of the synaptic weight change attained (Figure 2).^52^ This learning mechanism can then explain both the synaptic strength increase (potentiation) and decrease (depression). The former is caused by consecutive firing of the presynaptic neuron before the postsynaptic neuron, and the latter is caused by consecutive firing of the presynaptic neuron after the postsynaptic one. The smaller the time interval between two consecutive spikes, the larger the resulting synaptic weight change. Furthermore, learning is generally divided into two types regarding temporal retention: short-term potentiation (STP) or depression (STD), when a temporary change in synaptic strength takes place, and long-term potentiation (LTP) or depression (LTD), when a permanent change in synaptic strength occurs. The main factors that influence which one takes place are the frequency of neural activity—higher input frequencies have more chance of generating a permanent change than lower input ones—and the timing of the stimulus—the smaller the time separation between two consecutive stimuli, the higher the weight change and the chance of having permanent change induced.^16,50^ A particular form of STP often mentioned is paired-pulse facilitation (PPF), a phenomenon that takes place when a neuron is stimulated twice in a very short time interval in-between, such that the neuron’s response to the second stimulus is larger than that to the first.^53^ This response can be quantified by the PPF index (amplitude ratio between two consecutive spikes) as a measure used to study the control of neurotransmitter release at synapses.

## Resistive Switching Mechanisms

**Figure 3:**
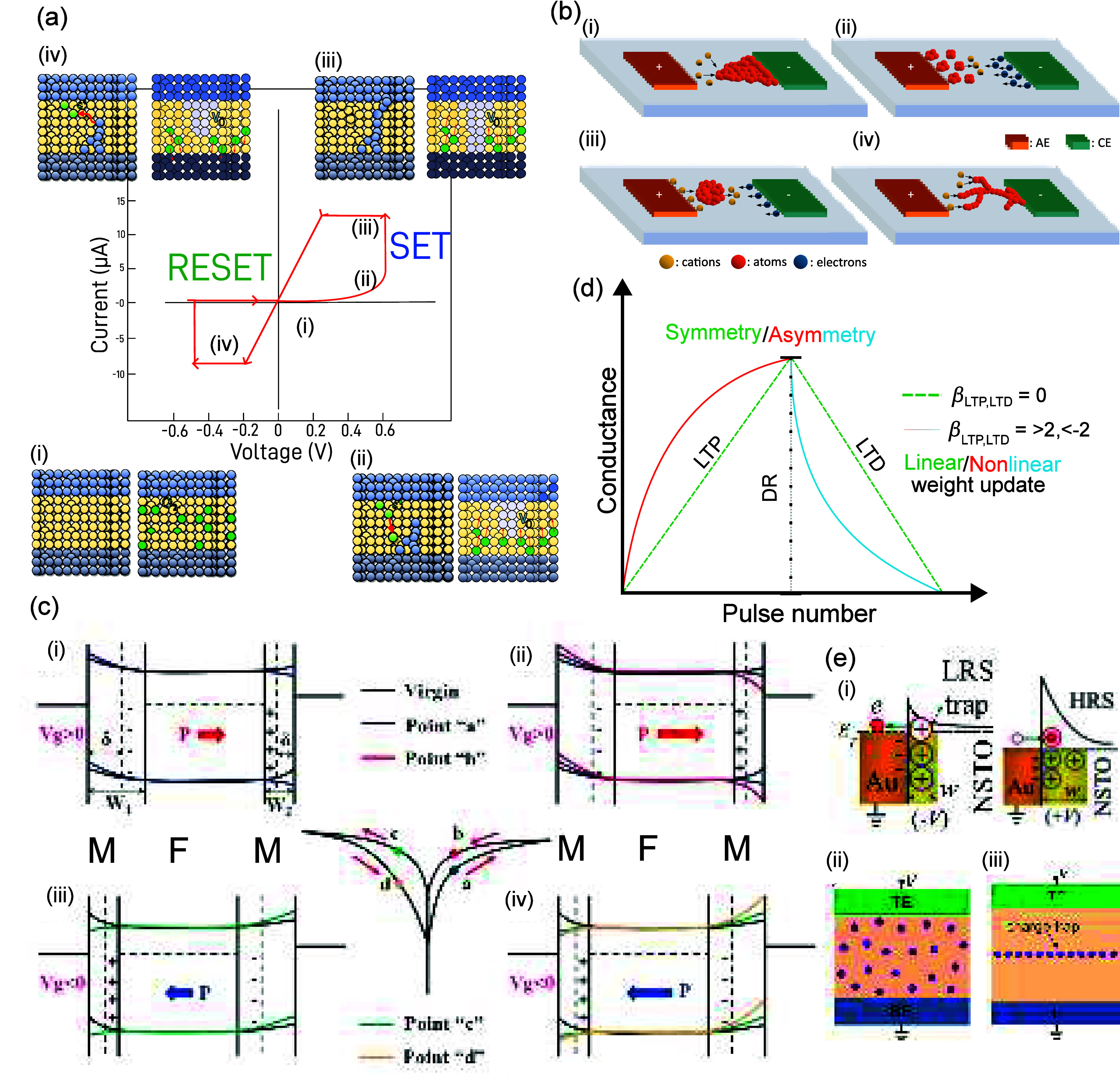
(a) Resistive switching dynamics of the redox-based stacks. In the initial state (i) the switching layer is insulating, (ii) but with applied voltage cations start to drift inside (left), while in the case of oxygen vacancies there is a drift to the opposite side of the positive biased electrode (right). (iii) At a certain threshold voltage bias (*V*_SET_), a filament is formed that bridges the two electrodes (ON state). This state is maintained until an opposite bias (*V*_RESET_), for the case of bidirectional switching, promotes the dissolution of the formed filaments, increasing the resistivity again (OFF state). (b) Metallic filament growth dynamics based on redox rate (Γ^*i*^) and cation mobility (μ): (i) high Γ^*i*^ and μ, (ii) low Γ^*i*^ and μ, (iii) high Γ^*i*^ but low μ, low Γ^*i*^ with high μ. (c) Ferroelectric switching process based on the modulation of the Schottky barrier height formed between an *n*-doped ferroelectric and two metal electrodes with different work functions (higher than that of the ferroelectric). Reprinted in part with permission from ref (54). Copyright 2015 John Wiley and Sons. (d) Relationship between conductance update and its nonlinearity and asymmetry. (e) Representation of charge traps originated at the (i) interface of the switching layer and the electrode, modulating the Schottky barrier’s height [Reprinted in part with permission under a Creative Commons CC BY-NC-ND 3.0 License from ref (55). Copyright 2013 Springer Nature] and charge traps inside the switching layer (ii) at random locations or (iii) introduced as single layers, modifying the conduction process. Reprinted in part with permission from ref (56). Copyright 2014 Elsevier.

Resistive switching (RS) can be defined as a resistance variation from a low (LRS or ON) to a high (HRS or OFF) resistance state and vice versa, upon the application of the appropriate stimulus, giving rise to a typical hysteretic current–voltage (*I*–*V*) curve [Figure 3(a)]. The change from LRS to HRS is called SET, and the inverse is called RESET. RS can be further categorized into volatile/nonvolatile and unipolar/bipolar, according to the retention time and voltage polarities, respectively.^57^ Depending on how the current changes under the applied bias (*I*–*V*), a memristor can be further categorized as digital or analog.^56,57^ In digital-type memristors, with the application of either *V*_SET_ or *V*_RESET_, an abrupt current change is seen at the transition from one state to another. In contrast, analogue-type memristors experience a gradual current increase/decrease with applied bias.

The resistance states and characteristic *I*–*V* shape depend on the inherent physical mechanisms and operation parameters. Different RS mechanisms were already proposed; however, here only the most relevant mechanisms for RS in MXenes will be reviewed: ion/photonic migration, diffusion and transport of Li^+^, ferroelectric switching, and charge trapping/detrapping.

### Ion Migration Switching

Resistive switching in ion migration systems usually relies on the formation of conductive filaments (CFs), connecting the two electrodes during the SET process (leading to the LRS). The rupture/dissolution of these CFs leads to HRS (RESET). An initial electro-forming step is sometimes needed at higher voltages to create defects in the switching layer, for ion-based RS.^57^ Ion migration RS can be further divided into cation- or anion-based, depending on the composition of the CFs.^56^

#### Cation-Based Switching

In cation-based memristors, the CFs are formed through electrochemical reactions of metallic ions, being therefore known as electrochemical metallization (ECM) memories.^58^ The SET and RESET processes, along with their basic electrochemical and physical phenomena, are illustrated in the left insets of Figure 3(a). In this example, an abrupt switching is seen as characteristic of filamentary ECM devices. This rapid conductance change can be explained by the electron tunneling processes occurring between the CF tip and the active electrode, which depends exponentially on the inverse of the tunneling distance.^57^ Therefore, a high current jump occurs mainly due to the conductance difference between electron tunneling in the HRS and metallic transport in the LRS.^16^ Regarding the contact type, depending on the applied current compliance, the distance between filament and electrode can either be a tunneling gap (as already described above) or galvanic (metallic-like).^57,59^ The conductive filament growth direction is directly linked to the contact type since ionic mobility will influence the shape and volume of the filament. A switching layer with higher ionic mobility will result in higher volume filaments, more prone to form galvanic contacts, and thus having more progressive SET and RESET. By contrast, switching layers with lower ion mobility are more likely to show abrupt RS.^57,60^ The direction of growth of conductive filaments in ECM stacks also depends on the mobility and redox rate of the metal cations. In typical ECM devices, growth occurs from the counter-electrode (CE) to the active electrode (AE) when there is high redox rate (Γ^*i*^) and cation mobility (μ) [Figure 3(b.i)]. In the opposite case (low Γ^*i*^ and μ), the growth occurs from AE to CE, as seen in Figure 3(b.ii). Besides these cases, the CF can also grow from CE to AE if only the mobility is high enough, while the low redox rate will lead to branched and random filaments [Figure 3(b.i–b.iv)]. Finally, they can also grow from the middle region to both electrodes if materials with high Γ^*i*^ and low μ are present [Figure 3(b.iii)].^11,56^

#### Anion-Based Switching

Anion migration normally happens with a stack of inert electrodes and an oxide layer containing, in the most common cases, sparse oxygen vacancies. Many VCM-based devices need a forming step to create more oxygen vacancies, to enable filament formation.^7,16^ As with ECM, the application of positive bias on one electrode will drift the positively charged oxygen vacancies toward the other, where they will accumulate and start to form a filament, which sets the device into LRS. With negative bias, the vacancies will drift back to recombine with their anion counterpart, thus breaking the filament and turning the device into HRS [right insets of Figure 3(a)].^16,56^ However, in this case, an important caveat should be mentioned: the direction of filament formation and the polarity of SET/RESET processes are dictated by the initial position of the vacancies.

### Ferroelectric Switching

Ferroelectric memristors can display RS due to the modulation of ferroelectric polarization with respect to an applied electric field,^61^ which can be related to different mechanisms, depending on their thickness. For thicker layers (>20 nm), electronic tunneling is negligible and the polarization-dependent switching effects are related to the modulation of the Schottky barrier, which is the most common case in current MXene studies.^62−64^ When a ferroelectric layer is in contact with a metal, an *n-* or *p-*type junction can be formed. In the case of the *n-*type junction, this leads to a rise in the conduction band energies of the ferroelectric layer at the interface, which then attracts positive charges to compensate for the bending of the bands [Figure 3(d.i)], forming a depletion layer. When no electric field is applied, the Schottky barrier is too high for charges to cross it, but when a positive bias is present at the biased electrode, negative polarization charges travel to it, while positive ones drift toward the grounded electrode [Figure 3(d.ii)]. Thus, the depletion layer becomes thinner and the Schottky barrier is lowered at the grounded electrode, allowing the flow of electrons, due to thermionic emission (in this case Schottky emission), as shown in Figure 3(d.ii).^65,66^ In contrast, with negative bias, positive bound charges accumulate at the top, enlarging the depletion field and the barrier [Figure 3(d.iii)], thus decreasing the conductivity of the device. At a certain bias, the ferroelectric polarization switches and the barrier at the grounded electrode is raised again, setting the device to the HRS [Figure 3(d.iv)].

### Electronic Migration

Resistive switching devices where traps are present can work in a purely electronic manner relying on the trapping/detrapping of charges, instead of the formation/rupture of metallic filaments.^56^ Depending on the polarity of the applied bias, the electrons injected into the switching layer can be trapped or detrapped. This can affect the injection barriers or the electronic transport mechanism, depending on the distribution of these charges in the switching layer.^16,56,67^ For interfacial charge traps [Figure 3(e.i)] formed at the junction of the switching layer and the electrode, their trapping state controls the height of the Schottky junction barrier, being responsible for the RS behavior in this case.^55,68^ Besides random displaced atomic level traps [Figure 3(e.ii)], originating from defects in the switching layer, sandwiched nanoparticles, nanotubes, or quantum dots in the switching layer have also been shown to introduce different charge trapping regions [Figure 3(e.iii)].^56,69,70^ For these, RS is explained by changing the inherent conduction process in the switching layer, generally through Coulomb repulsion of the trapped electrons (leading to HRS). Devices relying on this mechanism may present advantages in terms of stability, in relation to filamentary-type ones since no drastic structural changes occur during RS.

## Neuromorphic Properties of Memristors

The permanent increase or decrease in the electrical conductance is termed LTP and LTD, respectively, and is represented by a gradual change through the use of repeated pulsed stimulation. The use of these artificial synapses in learning applications, either online (software) or in the memory (hardware), is bound to specific performance parameters. These figures of merit (FoMs) will directly influence the learning accuracy and convergence^16,71^ and are generally accepted to be nonlinearity (NL), asymmetry (AS), dynamic range (DR), number of states (#G), and cycle-to-cycle (C2C) or device-to-device (D2D) fluctuations. For a systematic analysis of the FoMs from reported MXene-based memristors, here we focused on the NL, AS, and DR values. This choice encompasses the crucial parameters for in-memory learning, which are also frequently available in the reported studies. Even though most of the literature does not directly report the mentioned FoMs, these values can be extracted from the conductance curves. The conductance (*G*) and pulse number (*P*) are used to calculate the nonlinearity parameter^71^ using
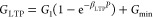
1for potentiation and
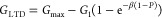
2for depression. In eqs 1 and 2, , β gives the linearity, *G*_max_/*G*_min_ are the maximum/minimum conductance values, and *P* is the normalized pulse number. From the NL parameter, we obtain the AS values using
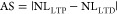
3Finally, the DR values can be calculated using
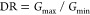
4Nonlinearity is a necessary feature for memories,^72^ in contrast with artificial synaptic emulation, where linear (NL_LTP,LTD_ < |1|) and symmetrical (AS < 1) conductance updates are wanted for accuracy. It is expected that an ideal artificial synapse, when symmetrical pulses (positive and negative) are applied, returns to the device initial conductance state^73^ [dashed line in Figure 3(d)]. Unfortunately, filamentary devices suffer from nonlinearity and asymmetry, which are inherent to the abrupt SET and RESET processes and uneven switching voltages and resistance ratios.^71,74,75^ Furthermore, the DR should be sufficiently high to avoid fluctuations arising from a very small separation between conductance states, which will degrade recognition accuracy. Nevertheless, for DR > 10 the accuracy saturates. The number of G levels is also positively correlated with the learning accuracy. However, a very large #G will lead to slow convergence especially when coupled with high linearity.^71^ In conclusion, low NL (<|1|) and asymmetry (<1), high DR (>10), and a moderate number of conductance states (64–512) should be sought-after in learning applications.

Filament growth dynamics directly influence the nonlinearity. In the SET process, the filament first grows until it bridges the two electrodes, being controlled by a drift process.^8,76^ With continued stimulus, the conductive filament will then thicken, but charge motion is now governed by diffusion.^16^ In the RESET process, as the filament is dissolved and the gap between the filament and electrode grows, it becomes more difficult for charges to migrate. In the SET (RESET) process, the conductance change (*ΔG*) rate will experience two stages: first, it will be lower (higher), and as it approaches *G*_max_ (*G*_min_), it will then be higher (lower). Furthermore, the dynamic range of the device is inherently tied to the switching layer resistivity, similar to the *R*_ON_/*R*_OFF_ ratio. Finally, the #G levels depends on the CF growth dynamics, with a more gradual growth allowing a larger number of G levels to be accessible.

## MXene-Based Memristors

### Fabrication

**Figure 4:**
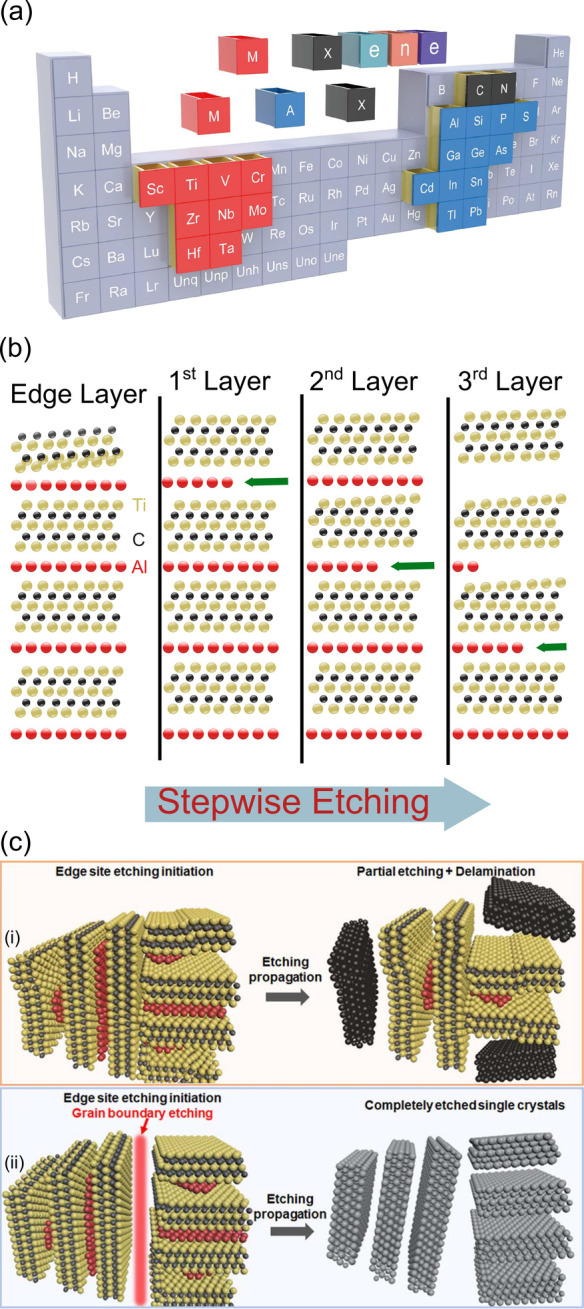
(a) Representation of the MAX and MXene elements in the periodic table. Reprinted in part with permission from ref (83). Copyright 2020 Elsevier. (b) Stepwise delamination of the Al layers on a MAX particle. (c) Grain boundary etching occurring in (i) LiF–HCl and (ii) pure HF methods. Reprinted in part with permission from ref (84). Copyright 2021 American Chemical Society.

The general MAX formula is M_*n*+1_AX_*n*_, where M is an early transition metal (Sc, Ti, Zr, etc.), A is an element from the periodic table groups IIIA or IVA (the most common is Al), X is nitrogen and/or carbon, and *n* = 1–3 [Figure 4(a)].^29^ The selective etching of the Al layer with hydrofluoric acid from Ti_3_AlC_2_, forming Ti_3_C_2_, originates a class of 2D metal carbides.^18^ For example, after the etching of the MAX phase, the Ti_3_C_2_ will have functional groups (depending on the etching method) such as −F or −OH on its surface; as such Ti_3_C_2_ is normally referred to as Ti_3_C_2_T_*x*_.^18^ The number of MXenes has grown significantly, both experimentally observed and theoretically predicted.^77−79^ This has also been observed in the memristor community with the introduction of V_2_C-based devices.^80−82^ This MXene is usually obtained from the V_2_AlC MAX phase, where an etching of the Al layer is performed similar to Ti_3_AlC_2_. Since this review is focused on MXene-based memristors, the following fabrication sections will explore the relevant steps for the explored RS MXenes (Ti_3_C_2_T_*x*_ and V_2_C) in their synthesis and deposition, including variations.

#### MXene Synthesis

The etching of Ti_3_C_2_T_*x*_, which was originally performed using HF (hydrofluoric acid), has since been achieved through multiple etchants.^19,29,84−88^ The ones usually used in memristive applications employ a mixture of hydrochloric and hydrofluoric acids (HF–HCl) or lithium fluoride with hydrochloric acid (LiF–HCl). These three routes (pure HF, HF–HCl, and LiF–HCl) affect the terminations on the etched MXene along with its physicochemical properties.^19,84,88^

**Figure 5:**
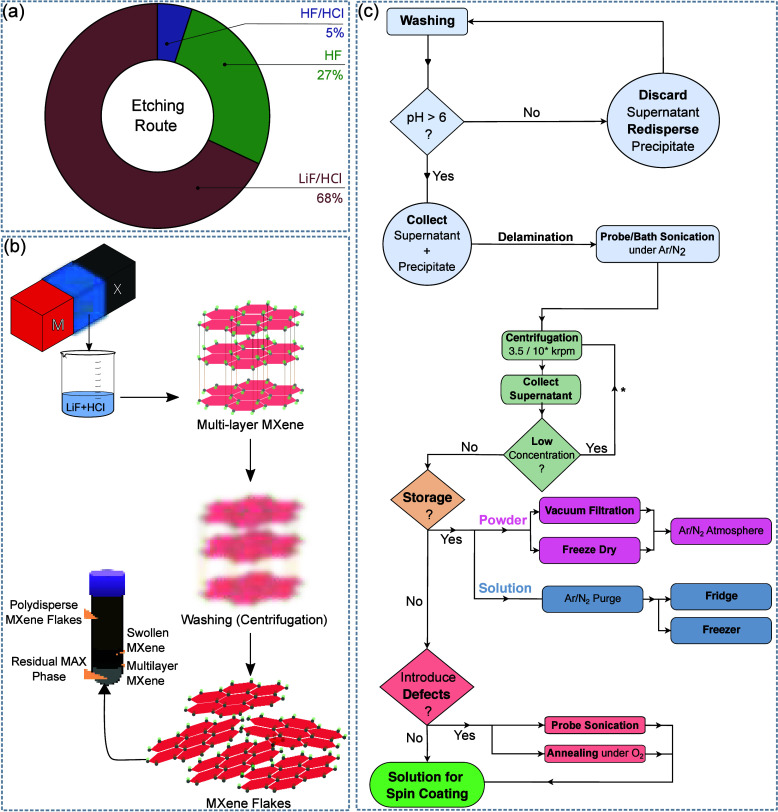
(a) Quantification of the etching method used (LiF–HCl,^30,31,62,63,80,81,90−98^ HF,^32,99−103^ HF–HCl^104^) for MXene-based memristors focused on neuromorphic properties. (b) MILD etching and delamination scheme resulting in MXene flakes (supernatant) and multilayer on top of residual MAX phase and other contaminants (precipitate). (c) Flowchart of the etching, delamination, and storage stages of Ti_3_C_2_T_*x*_ for memristive applications.

Each of the three etching methods mentioned displays clear advantages and disadvantages. The most obvious disadvantage comes from the hazardous handling of the pure HF method.^88^ Although this method can be made safer by using low HF concentration (5–10 wt %), while still being sufficient to etch the Al layer,^88^ it has so far not been used in memristive applications. A safer route to obtain Ti_3_C_2_T_*x*_ is to perform *in situ* HF formation from fluoride salts such as LiF,^88,89^ which can be combined with HCl to obtain an etchant with 3–5 wt % HF, lowering drastically the HF amount in the solution and avoiding handling pure HF. Beyond safety measures, pure HF and LiF–HCl etching differ in the way the etching process occurs. For both etchants, the exfoliation process starts equally with HF attacking Al layers step-by-step where, even though the MAX particle is surrounded by the etching solution, individual sheets are etched sequentially [Figure 4(b)].^84^ With LiF–HCl etching, grain boundaries are not broken [Figure 4(c.i)], resulting in an incomplete etching and lower MXene production yield.^84^ The best way to improve the yield here is to use pressureless sintered MAX phase powders without polycrystalline grains. In contrast, when the MAX particle is polycrystalline (the most common case), the pure HF method is able to break the grain boundaries of the MAX particle and thus expose the entire particle to the etchant [Figure 4(c.ii)], which leads to a complete etching. In relation to the mixed acid approach (HF–HCl), it has been shown to enable layer intercalation of water molecules which facilitates the delamination process, in comparison to the other methods.^19,105,106^ Pure HF etching is still a common choice for Ti_3_C_2_T_*x*_ memristive stacks, with HF–HCl being the least chosen and LiF–HCl being the most chosen, as quantified in Figure 5(a).

After exfoliation, it is necessary to wash the etched powder to stop the etching, preventing the etching of the other atoms. Since the interlayer interactions between MXene sheets are very strong, simple exfoliation through mechanical means, as typically used in other 2D materials, is not possible.^29^ Therefore, once the MXene powder has reached a pH > 6, an intercalation process must be used to expand the interlayer spacing of MXene to weaken the forces holding together the 2D sheets. Most MXenes can be intercalated by organic-based materials, such as dimethyl sulfoxide (DMSO),^107^ or tetraalkylammonium hydroxides, such as tetramethylammonium hydroxide (TMAOH).^108^ Metal cations can also be used to perform the intercalation.^29^

After the intercalation and subsequent washing of the intercalants, hand-shaking or sonication is enough to produce a colloidal suspension of single and multilayer MXene flakes. The choice of intercalant will influence the size, shape, and interlayer spacing of the produced flakes. For example, using LiCl results in larger flakes with fewer defects and shapes similar to those of the MAX particles.^88,109^ In contrast, the use of TMAOH produces flakes with higher defects and smaller size due to breakage during the delamination process.^108^ Regardless of the intercalant choice, the use of any type of mechanical vibration will reduce the size and create defects.^110^

As described, the etching and delamination processes have multiple steps and take considerable amounts of time (24–48 h for the etching process and about 4 h for the delamination). The complexity and time needed can be reduced by using the LiF–HCl method where, in the washing process (as pH rises), LiCl formed in the reaction will provide lithium ions which start to spontaneous delaminate the MXene layers, as depicted in the scheme of Figure 5(b).^89^ Therefore, the washing and delamination occur in the same step, although sonication can be required to obtain a larger yield of delaminated flakes. Nevertheless, by tuning the LiF to HCl amount, it is possible to produce an etchant with a higher HF content that provides a better etching yield and only needs hand-shaking isolation of single-flakes from the rest of the precipitated sediment. This method is commonly named minimally intensive layer delamination (MILD) and is usually used because it reduces the time and complexity of the process and provides large flakes with fewer defects.^21,88^ An optimized process for obtaining flakes by using the MILD route is presented in Figure 5(c). For the mixed acid approach (HF–HCl), since there are no large ions during the exfoliation process to intercalate between the MXene layers, a delamination step is required to introduce intercalating ions as in the pure HF method. Interestingly, some works reviewed here do not use intercalants and instead undergo very long ultrasonication steps of 10–12 h,^104,111,112^ which will naturally lead to small flakes with inherent defects.

For the case of V_2_C, the exfoliation energy of V_2_AlC is higher (0.205 eV Å^–2^) than that of Ti_3_AlC_2_ (0.164 eV Å^–2^),^113^ which means that it will be harder to etch to obtain V_2_C, leading to harsher exfoliating conditions. This was confirmed in the first method used to etch V_2_C based on pure HF at room temperature, which led to a large amount of impurities. Further tuning of the V_2_C etching method has revealed that a combination of NaF or LiF with HCl at 90 °C is best suited.^85^ A more recent study^85^ showed that LiF–HCl supplies V_2_C with higher purity and better electrochemical properties than NaF–HCl or HF etching due to the increased interlayer spacing caused by the intercalation of lithium ions. Additionally, it showed that etching V_2_AlC in a closed environment results in V_2_C with increased purity.

Some modifications of the synthesis process are being studied, which might be beneficial to the resistive switching mechanism. Contrary to the usual intention of enhancing the conductivity or charge storage capacity of MXenes for energy applications,^29,42,114,115^ in RS applications a reduction of the initial conductivity and the modification of the MXene’s flakes with the introduction of defects, oxidation, or surface terminations may be favorable in terms of performance. To obtain a high degree of oxidation and/or surface terminations, to decrease the conductivity or impart electronegativity to the surface, one can raise the etching temperature as performed by Sokolov et al.^99^ The higher etching temperatures form TiO_2_ particles^99^ and cause an incomplete Al removal. Better controlled oxidation approaches include the hydrothermal process of MXene solution (under O_2_)^116,117^ and annealing the MXene in air after deposition.^96^ Finally, flakes with a larger number of defects can be obtained with longer or more energetic sonication methods, such as probe sonication.

#### Stack Deposition

MXene flakes can be deposited using a variety of methods like spin- or spray-coating, rolling, and vacuum-assisted filtration. For the active layer in memristors, the best deposition method is spin-coating, since it provides high quality thin films with controlled thickness^88^ and a high degree of flake alignment.^118^ Due to the surface terminations inherent to the etching process, MXenes have a natural high hydrophilicity,^29^ which ensues a good compatibility with metallic surfaces typically used in memristor stacks (electrodes). Besides, it is possible to further optimize the deposition by functionalizing the substrate with positively charged molecules which attach to MXene terminations via electrostatic forces.^88^ Furthermore, the use of DMSO as a solvent provides better integration, density, and uniformity of flakes deposited in Si-based substrates.^31^ The MXene thickness can be controlled through the solution’s concentration (1–10 mg/mL)^119^ and the spinning parameters, as shown in Table 1. For instance, thin layers (<50 nm) are obtained with low flake density, high rpm, long spinning times, and single coating cycles. Generally, a solution with a high concentration of MXene flakes is better to optimize the coverage of the substrate.^120^ Controlling the thickness of the MXene layer is important since the conductivity increases with the thickness.^118^

**Table 1:** Spin-Coating Deposition Parameters Used for MXene-Based Memristors^a^

Stack	Spin Coating Time (s)	Spin Coating (rpm)	Annealing Time (min)	Annealing Temperature (°C)	Vacuum Dry	MXene Thickness (nm)
Al/Ti_3_C_2_T_*x*_/Pt^90^	n.r.	n.r.	-	-	Yes	150
Al/Ti_3_C_2_T_*x*_-TiO_2_ NF/Pt^101^	30	2500	60	80	-	500
Al/Ti_3_C_2_T_*x*_:Ag/Pt^104^	2× (n.r.)	2× (n.r.)	n.r.	n.r.	n.r.	100
Cu/Ti_3_C_2_T_*x*_/SiO_2_/W^30,31^	120	3500	-	-	-	2200
Cu/Ti_3_C_2_T_*x*_/PZT/Pt^63^	n.r.	n.r.	-	-	-	∼65
Cu/Ti_3_C_2_T_*x*_/BFO/Pt^62^	n.r.	n.r.	-	-	-	∼65
Ag/Ti_3_C_2_T_*x*_/SiO_2_/Pt^32^	60	500	-	-	-	50
Ag/AlO_*x*_/Ti_3_C_2_T_*x*_/ITO^93^	n.r.	n.r.	30	80	n.r.	n.r.
Ag/V_2_C/W^81^	60	1500	10	80	No	1500
Ag/V_2_C/TiO_2_/W^80^	60	1000	n.r.	n.r.	n.r.	n.r.
Au/LPE/Ti_3_C_2_T_*x*_/Pt^91^	20	800	20	90	N_2_	1300
Au/Ti_3_C_2_T_*x*_/Cu^98^	n.r.	n.r.	-	-	-	2.5
Pt/Ti_3_C_2_T_*x*_- TiO_2_/ITO^96^	2× 50	4000	60	200	-	n.r.

a n.r. stands for non-reported values and “-” where that parameter does not apply.

Other solution-based deposition methods have been explored, such as layer-by-layer (LbL),^121^ where amines help the compatibility of MXenes to the surface. Alternate dip coating of amines and MXene flakes is performed, leveraging the oppositely charged species effect, which enables control of the number of MXene layers deposited. This method can be an alternative to spin-coating for the control of the thickness in memristive stacks, as recently shown by Melianas et al.^92^ Flake transfer, commonly used with 2D materials, has also been used for artificial MXene synapses,^95^ by using a scotch tape to exfoliate vacuum-dried Ti_3_C_2_T_*x*_ until single-layer flakes were obtained. These flakes were transferred to a PDMS tape which, with the aid of an optical microscope, allowed its placement on the electrodes. This method offers great control over the amount of MXene layers present in the device. However, it allows only a limited deposition area.

#### Stack and Resistive Switching Characterization

As mentioned, the synthesis (etching choice, intercalant, degree of oxidation, etc.) of the MXene layer will greatly influence its properties, which, in turn, influence its interaction with the electrode materials and the RS behavior. Therefore, adequate characterization procedures are essential through the whole fabrication process, from MAX exfoliation to the integration of the MXene in the stack, to allow the fine-tuning of the properties.

During the etching process, different factors influence the flake’s size, shape, surface terminations, and interlayer spacing. Foremost, the MAX precursor should be analyzed under X-ray diffraction (XRD) to ensure that no contaminants are present.^40^

##### Interlayer Spacing

**Figure 6:**
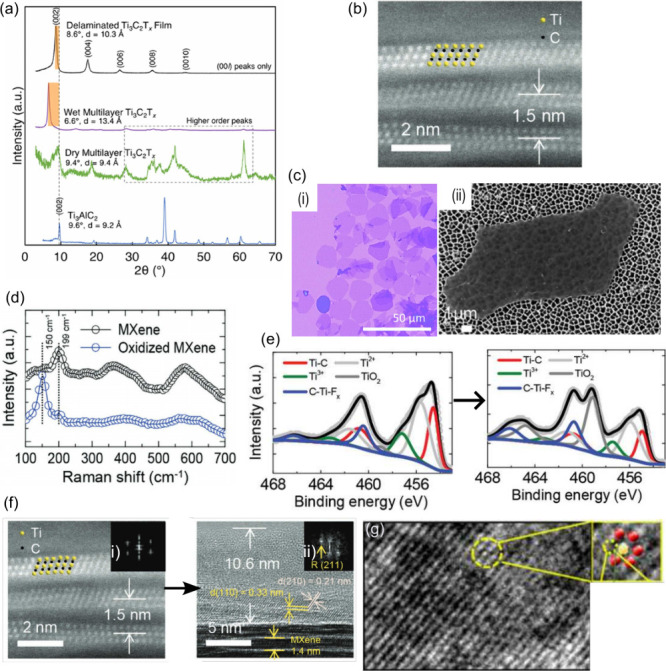
(a) XRD diffractogram of Ti_3_AlC_2_ and Ti_3_C_2_T_*x*_ samples at different steps of the fabrication process. Reprinted in part with permission from ref (40). Copyright 2021 Elsevier. (b) STEM image of pristine Ti_3_C_2_T_*x*_ flakes, showing the interlayer spacing. Reprinted in part with permission from ref (117). Copyright 2020 John Wiley and Sons. (c) Flake’s shape and size obtained with (i) optical microscopy and (ii) SEM. Reprinted in part with permission from ref (88). Copyright 2017 American Chemical Society and Reprinted in part with permission from ref (110). Copyright 2022, American Chemical Society. (d) Variation of the position and intensity of the Raman peaks, before and after oxidation. Reprinted in part with permission from ref (117). Copyright 2020 John Wiley and Sons. Evolution of oxidation (left to right) from pristine to severe oxidized Ti_3_C_2_T_*x*_ (20 and 360 min oxidation) analyzed through (e) XPS and (f) high-resolution STEM. Reprinted in part with permission from ref (117). Copyright 2020 John Wiley and Sons. (g) HRTEM image of an atomic vacancy found in Ti_3_C_2_T_*x*_ nanosheets. Reprinted in part with permission from ref (104). Copyright 2021 Elsevier.

XRD is extremely useful to probe the interlayer spacing. Since the MAX phases have a hexagonal structure (*p*_63_/*mmc* group), the crystal has two defining lattice parameters, *a* and *c*. The *c* parameter can be obtained from the (002) peak, which usually lies between 5 and 15°,^40,46^ so that the *d*-spacing will be given by *d* = *c*/2. After exfoliation, the A layer is removed, converting MAX to MXene with surface terminations and intercalant species now occupying the space where the A layer existed, changing the *c* lattice parameter. The resulting interlayer spacing can be measured in dried multilayer MXenes, using the (002) peak. This will correspond to the interlayer spacing of only the MXene sheets with the surface terminations, since all the intercalated water is removed. Using this as a reference, the XRD spectrum after delamination gives the interlayer spacing resulting from the delamination process [Figure 6(a)].^40^ It is important to note that the interlayer spacing of the deposited flakes will be different from that measured using XRD after delamination. Therefore, it is important to compare the (002) peak and thus the interlayer spacing before and after deposition. This can be further confirmed by advanced microscopy techniques such as high-resolution TEM (HRTEM)^112^ or scanning-TEM (STEM) that enable the visualization of this layer,^117^ as shown in Figure 6(b).

##### Flake Size and Defects

After a solution of MXene flakes is obtained, it is important to observe their shape and size. For flakes larger than 1 μm, optical microscopy (OM) provides very good contrast just by drop-casting a MXene solution in a Si/SiO_2_ substrate, as shown in Figure 6(c.i).^110,122^ Scanning electron microscopy (SEM) can also be used with anodic aluminum oxide membranes^110^ that provide enough contrast to visualize down to single-layer MXene flakes, as seen in Figure 6(c.ii). This method offers high quality images of the shape and size of the flakes, although it can lead to unwanted damage in larger flakes.^88,110^ SEM resolution further enables the observation of pinhole defects and edge oxidations which are not detectable using XRD or OM.^40^ The size distribution can also be estimated through SEM or dynamic light scattering (DLS). After depositing the MXene layer in the substrate for the memristive stack, OM [Figure 6(c.i)] and SEM [Figure 6(c.ii)] allow the observation of the density of flakes covering the substrate and their disposition.

##### Flake Thickness

The best technique to obtain the thickness of the MXene flakes is atomic force microscopy (AFM). Note that, due to naturally intercalated molecules (such as water), the AFM measurement will be slightly larger than the actual thickness.^40,95,98,110^

##### Oxidation

Flake oxidation can be assessed by intensity changes and the appearance of peaks in Raman or XRD techniques. The Raman spectra of MXenes are composed of in-plane (E_g_) and out-of-plane vibrations (A_1g_). In Raman, after oxidation, the peaks at around 205 and 723 cm^–1^, attributed to the out-of-plane vibrations of Ti, C, and O atoms, almost disappear. At the same time, a peak at 150 cm^–1^ will appear due to the E_g_ mode of the oxidized layer (TiO_2_), as represented in Figure 6(d).^41,96,117^ Note that usually the Raman spectrum is not acquired in a vacuum, and therefore, natural oxidation can be present. In the case of XRD, peaks will appear at around 25.3° and 50.5°, corresponding to the TiO_2_ anatase phase, and at 27.3°, 36.1°, and 41.2° for the rutile one.^96,123^ However, only relative information on the oxidation layer can be retrieved, by comparing with another sample fabricated with the same parameters. Therefore, other techniques should be used to extract quantitative information. X-ray photoelectron spectroscopy (XPS), due to its low penetration depth (∼10 nm), can probe the surface of flakes. XPS spectra of Ti_3_C_2_T_*x*_ around 450–470 eV enable the observation of the core-level energy spectrum of Ti 2p, which presents multiple peaks from Ti–C (Ti^+^), Ti–X (Ti^2+^), Ti_*x*_O_*y*_ (Ti^3+^), and TiO_2_ (Ti^4+^) at around 454.6, 455.5, 456.7, and 458.5 eV, respectively, for the Ti 2p_3/2_ component.^96,116,117,124^ Since oxidation of the surface of Ti_3_C_2_T_*x*_ is formed by the oxidation of Ti^+^, Ti^2+^, and Ti^3+^ into TiO_2_, the growth of the Ti^4+^ peak together with the decrease of the other three peaks is observed as oxidation increases, in the XPS spectra of Figure 6(e). When paired with ion-beam etching, XPS depth profiling provides a quantitative measurement of the oxidation layer depth by comparing the relative contents of Ti^3+^ to Ti^4+^.^116^

Another technique commonly used is TEM where, due to its (also) low penetration depth, the complete visual observation of the MXene surface can be performed. A full resolution image of the oxidation layer can be obtained with STEM [Figure 6(f)], enabling the direct measurement of its thickness.^40,99,116^ Besides measuring the oxidation layer, STEM allows the observation of the atomic structure, changing with oxidation from hexagonal (pristine) to a different crystal structure [rutile phase; insets of Figure 6(f.i) and (f.ii), respectively].^116^

##### Surface Terminations

Surface terminations play a crucial role in tuning the MXene electrical properties,^34,39^ which will influence the RS behavior. Therefore, it is important to resort to different techniques to tune the type and amount of terminations under different etching conditions. Raman spectroscopy can be used, since in E_g_ vibration groups there is a whole region (230–470 cm^–1^) solely affected by surface atoms.^125^ In the case of milder etching routes (MILD and HF–HCl) there is a prominence of the −O component, while for a pure HF method there is a dominance of the −OH and −F terminations.^125^ However, Raman vibrations are affected by a multitude of factors like if the flakes are wet (have intercalated water) and if they are delaminated (interlayer spacing), among others.^40,125^ Therefore, it is advisable to couple different techniques. Another aspect to consider is that, by knowing the plasmon resonance, better Raman spectra can be acquired with the appropriate laser wavelength.^125^ For the specific case of Ti_3_C_2_T_*x*_, it has a plasmon resonance peak at around 780 cm^–1^, and therefore, using a 785 nm laser will result in higher definition. Since XPS not only reveals the elements present but also the bonding between different elements, it is a useful technique to investigate the surface groups.^40,126^ In particular, the binding energy intensities of commonly adsorbed ions will show the presence and relative amount of specific surface terminations.^98,99^ The Ti 2p region of the XPS spectrum, besides being very valuable to analyze Ti_3_C_2_T_*x*_ oxidation, also provides peak information on −O and −F surface species. The O 1s zone contains the peaks for surface terminations such as −O and −OH, while the F 1s zone shows −F terminated peaks.^39,96,98,127^ STEM can be used to analyze surface groups, although due to their randomness and low weight, they are very difficult to detect (except for −Cl terminations).^40^ Nevertheless, when coupled with ultrahigh-resolution electron energy loss spectroscopy (EELS), it allows a deeper analysis by probing both the nature of the surface groups and the site they occupy on the surface. Therefore, it is possible to study the relative quantity of surface defects (−O, −F, −OH, or −Cl) produced by different etching routes.^40,128^ Since both EELS and XPS are realized in a vacuum, this can lead to desorption of some surface terminations, which must be taken into account when interpreting the data.^40^ Finally, the pair distribution function analysis (PDF) technique is one of the most powerful to extract a wide array of MXene structure data, especially about surface groups.^40^

As the mechanism behind RS in MXenes is still not completely understood, it is important to identify the useful techniques able to reveal the different factors involved, such as surface terminations, interlayer spacing, oxidation state, and atomic vacancies. As examples, XPS can be used to indirectly determine the amount of oxygen vacancies in the flakes, as shown in Figure 6(g),^104^ while TEM can probe Ti vacancies.^90^ Cross-section TEM coupled with EDS can be used to confirm filamentary RS.^98^ Finally, Kelvin probe force microscopy (KPFM) can be used to study the electrostatic potential and EELS to determine the distribution of atomic vacancies.^103^

### Resistive Switching in MXenes

**Figure 7:**
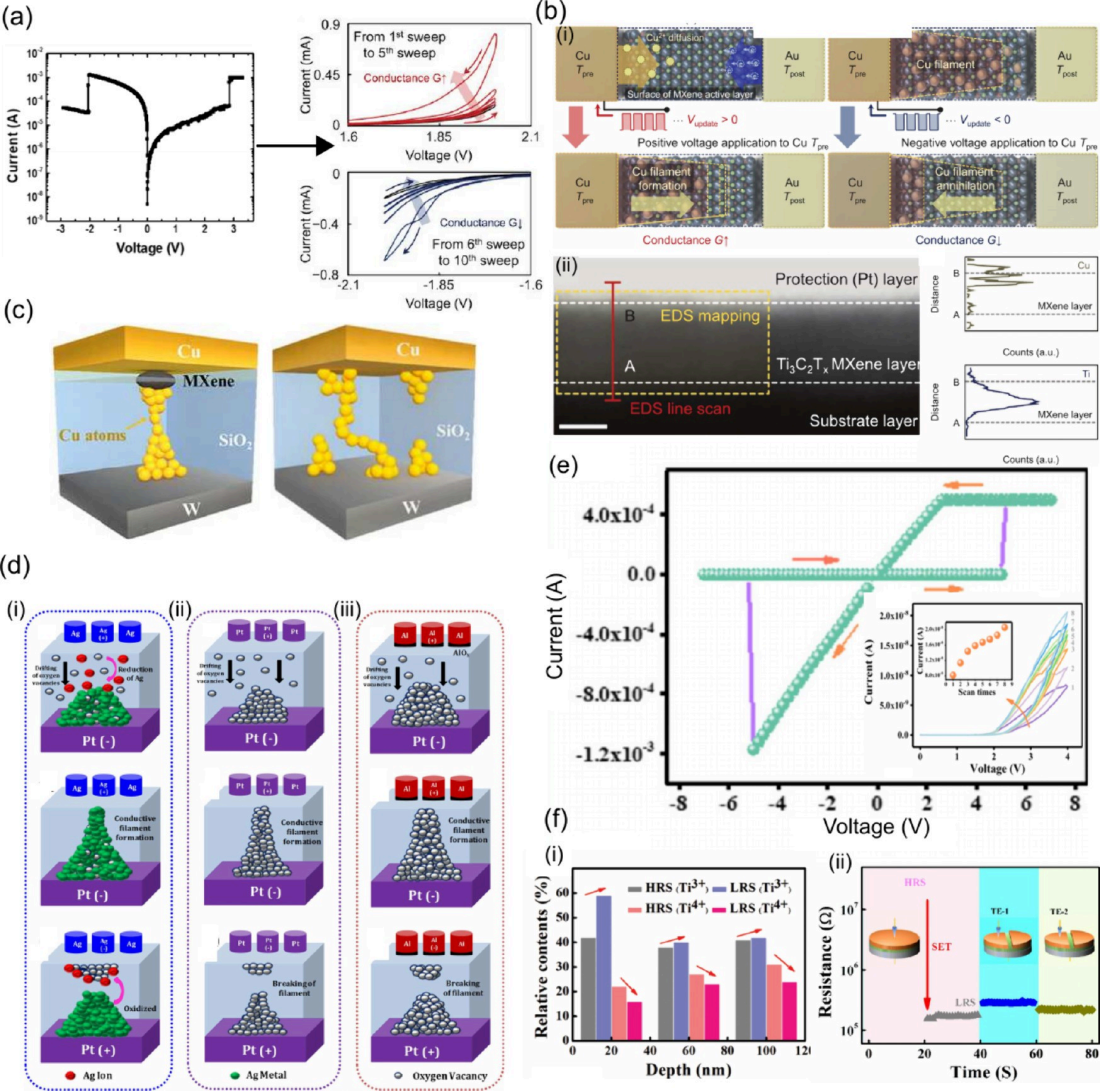
(a) Abrupt conductance change in the forming process of Cu/Ti_3_C_2_T_*x*_/Au, followed by a gradual conductance change with subsequent stimulus. Reprinted in part with permission from ref (98). Copyright 2021 John Wiley and Sons. (b) Cu filament dynamic of a planar Cu/Ti_3_C_2_T_*x*_/Au stack, where (b.i.) the surface terminations help the growth of the metallic Cu filament to be formed on top of the MXene flakes and (b.ii.) the EDS confirmation of this growth. Reprinted in part with permission from ref (98). Copyright 2021, John Wiley and Sons. (c) Effects of the insertion of the Ti_3_C_2_T_*x*_ layer in a Cu/SiO_2_/W stack, where the Ti vacancies promote the ordered growth of metallic filaments. Reprinted in part with permission from ref (129). Copyright 2019 AIP Publishing. (d) Growth dynamic of (Ag, Al, and Pt)/Ti_3_C_2_T_*x*_/Pt. Reprinted in part with permission from ref (100). Copyright 2021 American Chemical Society. (e) Demonstration of voltage-dependent digital and analog behaviors in a VCM-based stack. Reprinted in part with permission from ref (95). Copyright 2022 American Chemical Society. (f) XPS line profiling evolution of Ti^3+^ and Ti^4+^ relative content with Al/Ti_3_C_2_T_*x*_/Pt device resistance state and active layer depth. The inset shows the cutting of the TE in half, proving the resistance remains the same for both halves, therefore dismissing the formation of CF. Reprinted in part with permission from ref (90). Copyright 2019 John Wiley and Sons.

Most MXene-based memristors with an ECM mechanism rely on filament switching dynamics, with some needing a forming step. This can be observed, for example, in the lateral memristor stack of Cu/Ti_3_C_2_T_*x*_/Au^98^ seen in Figure 7(a). Reports without forming usually rely on MXene as a resistive layer,^81^ which is not very insulating, or MXene doping with Ag^+^, so that cations do not need to cross the electrode/switching layer barrier.^104^ Then, there are some works that inhibit the forming step using VCM, by oxidizing the MXene layer, increasing its porosity and vacancies reservoir, along with choosing electrodes that also contribute to this reservoir.^96,101^ Finally, there are also ferroelectric heterostructures, where the lattice mismatch lowers the interfacial barrier for ions to cross, together with the help from the ferroelectric polarization effect.^62,63^

The choice of the active electrode is crucial since it generally takes part in the RS mechanism. Ag or Cu electrodes are commonly used in ECM-based devices because of their low standard electrode potential (conferring high redox rate) and of their low Gibbs free energy oxide formation (enabling high cation mobility).^56^ This is reflected in the choice of the active electrode on ECM MXene-based memristors for artificial synapse emulation, as shown in Table 2. The only reported exception uses Al because the switching layer is already doped with Ag nanoparticles.^104^

**Table 2:** MXene-Based Memristive Structures for Artificial Neural Networks^a^

Structure	RS Mechanism	Conduction Mechanism	Electroforming
Al/Ti_3_C_2_T_*x*_/Pt^90^	Electronic Migration	TAT	Yes (n.r.)
Al/Ti_3_C_2_T_*x*_:Ag/Pt^104^	ECM	n.r.	No
Al/Ti_3_C_2_T_*x*_-TiO_2_ NF/Pt^101^	VCM	SCLC	No
Cu/Ti_3_C_2_T_*x*_/SiO_2_/W^31^	ECM	n.r.	n.r.
Cu/Ti_3_C_2_T_*x*_/SiO_2_/W^30^	ECM	n.r.	6 V
Cu/Ti_3_C_2_T_*x*_/Au (lateral)^98^	ECM	n.r.	3 V
Cu/Ti_3_C_2_T_*x*_/PZT/Pt^63^	FE + ECM	Schottky Emission	No
Cu/Ti_3_C_2_T_*x*_/BFO/Pt^62^	FE + VCM	SCLC	No
Ag/Ti_3_C_2_T_*x*_/SiO2/Pt^32^	ECM	n.r.	1 V
Ag/Ti_3_C_2_T_*x*_-TiO_2_/Pt^99^	ECM	n.r.	20 V
Ag/Ti_3_C_2_T_*x*_-TiO_2_ NF/Pt^101^	ECM	SCLC	No
Ag/AlOx/Ti_3_C_2_T_*x*_/ITO^93^	n.r.	n.r.	n.r.
Ag/V_2_C/W^81^	ECM	Schottky Emission	No
Ag/V_2_C/TiO2/W^80^	ECM	SCLC	n.r.
Au/LPE/Ti_3_C_2_T_*x*_/Si^91^	Li^+^ Diffusion	n.r.	n.r.
Pt/Ti_3_C_2_T_*x*_/Pt^95^	VCM	SCLC	No
Pt/Ti_3_C_2_T_*x*_-TiO_2_/ITO^96^	VCM	Ohmic	No

a Non-reported values are represented as n.r., and FE stands for ferroelectric.

The growth direction of filaments depends largely on the type of electrode and the switching medium. The AE to CE filament growth direction [Figure 3(b.ii)] is uncommon with Ag or Cu electrodes because they generally have high mobility, regardless of the medium, even if their redox rate can vary significantly. Thus, to achieve this type of growth, it is necessary to reduce the mobility of the metal cations. Park et al. leveraged the surface terminations of the Ti_3_C_2_T_*x*_ layer, by building a lateral Cu/Ti_3_C_2_T_*x*_/Au structure.^98^ In this case, when a stimulus is applied, the Cu electrode injects Cu^2+^ into the surface of the MXene layer. Due to the high electric flux density at the surface, the ions will preferentially grow along this region. As they are injected on the MXene surface, the surface terminations will slow down the movement of Cu^2+^, piling them near the AE, so that the filament growth occurs from the AE to CE [Figure 7(b.i)], as confirmed by EDS mapping [Figure 7(b.ii)]. The filament in this case grows along the MXene layer surface with the help of the surface terminations, which can enable more conductance states to be formed.

Besides the choice of electrodes, the switching layer plays a major role in dictating the switching mechanism and its repercussions on device performance. A clear example of this was shown by introducing Ti_3_C_2_T_*x*_ in SiO_2_-based structures. SiO_2_ memristors show CF-based RS and have been extensively studied due to their low-cost fabrication and CMOS compatibility.^130^ Nevertheless, these devices still show low stability and large parameter distribution.^94^

Knowing that 2D materials had been used to improve a memristor’s performance,^14,131^ Tong’s group inserted Ti_3_C_2_T_*x*_ nanosheets in traditional SiO_2_-based stacks, namely, TiN/Cu/SiO_2_/TiN,^94,132−134^ Cu/SiO_2_/W,^30,31,129^ and Ag/SiO_2_/Pt.^32^ The observed large improvement in cycle-to-cycle reproducibility was attributed to the reduction of the randomness of CF formation due to their growth occurring through the ordered location of the MXene nanostructures, where the negatively charged electrons from the MXene matrix facilitate the reduction of Ag/Cu ions^132^ and Ti vacancies captured and reduced them, enforcing filament nucleation along vacancies.^32,133,134^ The Cu and Ag electrodes generally have high mobility in the switching layers due to their standard Gibbs free energy formation of oxides. However, the disordered filament growth in the SiO_2_ layer shown in Figure 7(c) indicates that the redox rate of Ag/Cu ions is low, as depicted in Figure 3(b.iv).

Even though the most common RS in MXene stacks is cation-based, due to MXene’s advantage of capturing cations through their negatively charged Ti vacancies, there are studies that explore VCM. For filamentary VCM, it is important to have a rich source of vacancies, which can be achieved by tuning the switching layer, the choice of electrodes, or the introduction of oxide layers.^135^ When developing VCM devices, the mentioned properties of Ag/Cu electrodes can be detrimental. Khot et al.^100^ studied stacks of (Ag, Pt, Al)/Ti_3_C_2_T_*x*_/Pt. All structures showed similar *V*_SET_/*V*_RESET_ values, although the SET and RESET processes were not equal, especially with Ag showing a fluctuating RS behavior. This was explained by the existence of both VCM and ECM mechanisms, with the biased electrode being reduced and forming Ag^+^ (ECM), while at the same time oxygen vacancies from Ti_3_C_2_T_*x*_ also contribute to RS (VCM). Therefore, two competing RS mechanisms occur, with the HRS region being dominated by hopping phenomena [due to the residual CF; Figure 7(d.i)]. Interestingly, a different Ag/Ti_3_C_2_T_*x*_/SiO_2_/Pt^32^ stack did not demonstrate this mixed switching. On the other hand, both Pt and Al devices rely only on oxygen vacancy migration [Figure 7(d.ii and d.iii)], with a space-charge-limited-current (SCLC) transport in the HRS region, and thus have a more stable switching behavior without competing effects. Better performance and stability were achieved with Al, due to the formation of an AlO_*x*_ layer during Al deposition, which acts as an additional reservoir of oxygen vacancies, contributing to a thicker and more stable filament.

Zhang et al. studied VCM Pt/Ti_3_C_2_T_*x*_/Pt^95^ and showed the effect of low vacancy content in the switching layer. In this case, the oxygen vacancies are provided by the partially oxidized TiO_*x*_ naturally formed under ambient conditions (confirmed by XPS analysis). They show both digital and analog switching where for a higher operational voltage (7 V) a fully formed CF appeared very rapidly, but limiting the voltage up to 4 V, gradual conductance changes were observed [Figure 7(e)].

One example that differs from the ion-migration ones is the Al/Ti_3_C_2_T_*x*_/Pt stack,^90^ where a purely electronic migration takes place through a trap-assisted tunneling (TAT) conduction mechanism of electrons for both LRS and HRS. TAT occurs when the switching layer is rich in trapping sites (e.g., oxygen vacancies or defects such as Ti vacancies in Ti_3_C_2_T_*x*_), that assist in the tunneling of electrons from AE to CE.^16,136^ With a forming step, oxygen vacancies are formed in the MXene layer, along with some oxidation of the titanium ions, probably due to the thermophoresis effect caused by Joule heating. These defects aid the movement of electrons (the trap energy is decreased in the LRS). This was confirmed by XPS of both LRS and HRS; as the amount of oxygen vacancies is tied to the electrical resistance, the Ti^4+^ amount will differ depending on the resistive state^90^ [Figure 7(f.i)]. The formation of CFs, in this device, was experimentally dismissed by setting the device to the LRS and then cutting the top electrode in half, where it was found that the resistance of both sections was identical [Figure 7(f.ii)].

#### Modifications of MXene Layers for RS Tuning

The fine-tuning of the active layer and the electrodes has been an ongoing effort to improve performance. As mentioned, the ease of preparing Ag electrodes to be electrochemically dissolved will degrade the performance over time. Wang et al.^104^ replaced Ag with Al and doped the Ti_3_C_2_T_*x*_ switching layer with Ag nanoparticles. Since Ti_3_C_2_T_*x*_ already has negatively charged Ti vacancies [Figure 6(g)], the positive Ag^+^ formed under an applied bias will be attracted to these sites. These have a higher current density (as confirmed by finite element analysis simulations), thus serving as a driving force for the Ag filament formation.

**Figure 8:**
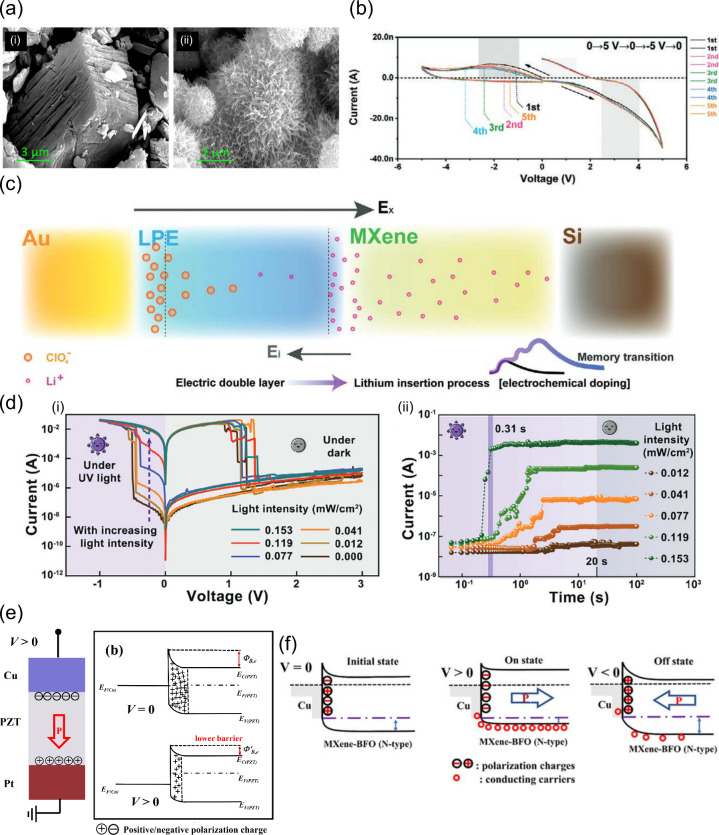
(a) SEM image of morphological effects comparison between Ti_3_C_2_T_*x*_ (i) pristine flakes and (ii) alkalization-treated ones. Reprinted in part with permission from ref (101). Copyright 2023 Elsevier. (b) Open-loop *I*–*V* curves for the Au/LPE/Ti_3_C_2_T_*x*_/Si device and (c) representation of the intercalation of Li^+^ in the MXene layer. Reprinted in part with permission from ref (91). Copyright 2021 John Wiley and Sons. (d) Effects of UV light stimulation on the switching properties of the ITO/Ti_3_C_2_T_*x*_-ZnO/Al stack. (d.i) Decrease of SET voltage with increasing light intensity; (d.ii) current increase over time under light stimulation for various intensities. Reprinted in part with permission from ref (103). Copyright 2021 John Wiley and Sons. (e) Switching (Cu/Ti_3_C_2_T_*x*_/PZT/Pt) aided by a ferroelectric effect of band bending with the application of positive bias, lowering the energy barrier for Cu ions to enter the Ti_3_C_2_T_*x*_ layer. Reprinted in part with permission from ref (63). Copyright 2022 Elsevier. (f) Energy band diagram demonstrating the variation of the band’s height from the ferroelectric BFO, which enables an easier crossing of the vacancies to the Ti_3_C_2_T_*x*_ layer, for the Cu/Ti_3_C_2_T_*x*_/BFO/Pt stack. Reprinted in part with permission from ref (62). Copyright 2023 Elsevier.

Since the commonly used MXenes (Ti_3_C_2_T_*x*_ and V_2_CT_*x*_) possess high conductivity, this can limit the RS range or even lead to short-circuit. This has been bypassed by using MXene layers in conjunction with dielectrics.^30−32,62,63,80,91,93^ For MXene as the only active element, deliberate oxidation naturally reduces the conductivity, resulting in improved performance. Sokolov et al.^99^ used an unusually high etching temperature (65 °C) to produce partially oxidized Ti_3_C_2_T_*x*_ nanosheets. The oxidation and abundant surface terminations obtained reduced the conductivity of the flakes and increased the electronegativity at their surface, respectively. This enhanced the diffusion of Ag cations to form a conductive filament, enabling volatile RS, while in the unoxidized case, no RS was achieved (short-circuit). However, this oxidation leads to an incomplete removal of Al atoms and porous nanosheets, which create a short-circuit between the electrodes, requiring the addition of a binder (cellulose). Furthermore, high electroforming voltages (20 V) were needed and long-term plasticity was not achieved. To obtain a more uniform oxidation coverage, Feng et al.^96^ applied a higher annealing temperature for longer time (200 °C for 1 h) after Ti_3_C_2_T_*x*_ deposition (Table 1). The partially oxidized MXene layer showed an improved resistance ratio and lower operating voltages than the one without oxidation. This improvement was explained by both the higher quantity of oxygen vacancies due to the formation of TiO_2_ in the active layer and the use of indium tin oxide (ITO) as an electrode and the presence of more surface terminations that raise the electronegativity of the nanosheets. When combined, the additional oxygen vacancies promote thicker filament formation and the surface terminations promote vacancy diffusion in the switching layer. Khot et al.^101^ employed both morphology changes and oxidation, through the simultaneous oxidation and alkalization of Ti_3_C_2_T_*x*_. This process resulted in Ti_3_C_2_T_*x*_-TiO_2_ with a nanoflower morphology [Figure 8(a)] that increased the reservoir of oxygen vacancies (as confirmed by XPS) and a porous structure that allowed enhanced ionic/electrical transport due to the increased surface-to-volume ratio. The use of Al as an electrode allowed the formation of a thin AlO_*x*_ layer that added even more oxygen vacancies. These factors contributed to the formation of vacancy-based CFs without electroforming, with low and almost symmetric SET/RESET voltages (0.68 V/–0.53 V).

Taking inspiration from solid-state batteries, neuromorphic systems based on doping and ion intercalation using electrolytes have also been greatly explored. Their basic operation lies on the ionic dynamics occurring in the electrolyte layer, where the reservoir of ions will move toward the switching layer (or the channel material in 3-terminal devices).^16,137^ As the ions reach the switching layer/gate, they form an electric double layer (EDL), which, in turn, changes the conductance. Since this EDL is very thin, a very large electric field will be generated, enabling operational voltages much lower than those of traditional filamentary-based devices. Wei et al.^91^ developed an electrolyte-based stack with two terminals, using a lithium polymer electrolyte (LPE) and a MXene layer for ion storage (Au/LPE/Ti_3_C_2_T_*x*_/Si). Interestingly, this device does not have memristive behavior, as shown by the open *I*–*V* scans [Figure 8(b)], but it displays neuromorphic properties. Under an applied bias, the solid lithium polymer provides Li^+^ to the MXene layer surface, forming an EDL at the LPE/MXene interface and switching the device to the ON state. However, by removing the stimulus, the ions spontaneously diffuse back to the LPE and thus destroy the EDL, turning the device into the OFF state. This inherent volatility is typical of electrolyte-gated devices based on EDL processes. However, it is known that MXene is an excellent ion storage material due to its low ionic diffusion barrier and presence of trapping sites.^29,89^ Therefore, with repeated stimulus, some Li^+^ can intercalate into the MXene structure, so that the time it takes for their return to the original position increases, leading to potentiation [Figure 8(c)].

Wang et al.^103^ have recently reported the doping of Ti_3_C_2_T_*x*_ with ZnO nanoparticles. Under electrical stimulation, the MXene-ZnO layer shows enhanced stability with lower operation voltages (−0.31 V/1.3 V) and higher resistance ratio (≃10^4^). In this case, XPS points toward the formation of oxygen-vacancy-based CFs. The same device was also able to perform SET under optical stimulation (λ = 365 nm), while an electrical stimulus was necessary for the RESET [Figure 8(d.i)]. Further testing showed that the reduced *V*_SET_ with increasing light intensity was explained by a spontaneous SET operation under sufficient optical stimulus [Figure 8(d.ii)].

Lastly, MXenes have been used to improve the volatility of ferroelectric switching.^62,95^ A layer of Ti_3_C_2_T_*x*_ between a Cu electrode and the lead zirconate titanate (PZT: PbZr_*y*_Ti_1–*y*_O_3_) switching layer was shown to improve the RS properties compared with the original Cu/PZT/Pt stack,^63^ by lowering the Cu^2+^ diffusion energy barrier, and also due to the compatibility between the PZT and MXene because of the surface terminations of the latter. The switching mechanism is dominated by metallic CFs (confirmed by temperature-resistance tests) with a weak ferroelectric effect. This can be explained by the fact that positively biasing the Cu electrode makes the polarization at the PZT layer point toward Pt, leading to a reduction of the depletion layer at the MXene/PZT interface, lowering the Schottky barrier. This allows the migration of Cu^2+^ ions, their reduction, and ultimate formation of the conductive filament connecting the Pt and Cu electrodes, setting the device to the ON state [Figure 8(e)]. Zhang et al.^62^ used barium ferrite (BFO: BaFe_12_O_19_), with weak ferroelectric behavior,^138,139^ in conjugation with Ti_3_C_2_T_*x*_. By exploiting the low coercive field of BFO together with the Ti_3_C_2_T_*x*_/BFO interface energy,^140^ two regimes of volatile and nonvolatile RS were achieved, depending on the *I*_CC_ range. With lower *I*_CC_, the switching is purely ferroelectric and volatile, with a lowering of the Schottky barrier at the interface where the ferroelectric polarization points to, leading to the LRS. However, the low coercive field of the BFO layer allows for the rapid reversal of its polarization (volatile mode). Increasing *I*_CC_ from 10 to 100 μA, the drift of oxygen vacancies in the BFO layer starts to also play a role in the modulation of the Schottky barrier. Since MXene/BFO is considered as an *n*-type contact [Figure 8(f)], the positive bias attracts positive bound ferroelectric charges to the Cu/MXene–BFO interface, inducing the drift of existing oxygen vacancies in the BFO layer. Additionally, lattice mismatch between the BFO layer and the Ti_3_C_2_T_*x*_ lowers the formation energy of oxygen vacancies and reduces the corresponding migration barrier. All of these factors contribute to a stronger resistive switching mechanism that overcomes the depolarization field, making it nonvolatile.

### Neuromorphic Applications in MXene-Based Devices

Different synaptic functions were already mimicked by memristors, such as short-term potentiation and depression (STP/STD), long-term potentiation and depression, spike-timing-dependent plasticity, or spiking-rate-dependent plasticity (SRDP). An attempt to evaluate their resemblance with biological plasticity resorts to the linearity and asymmetry parameters.^73^

#### Short-Term Potentiation and Depression

**Figure 9:**
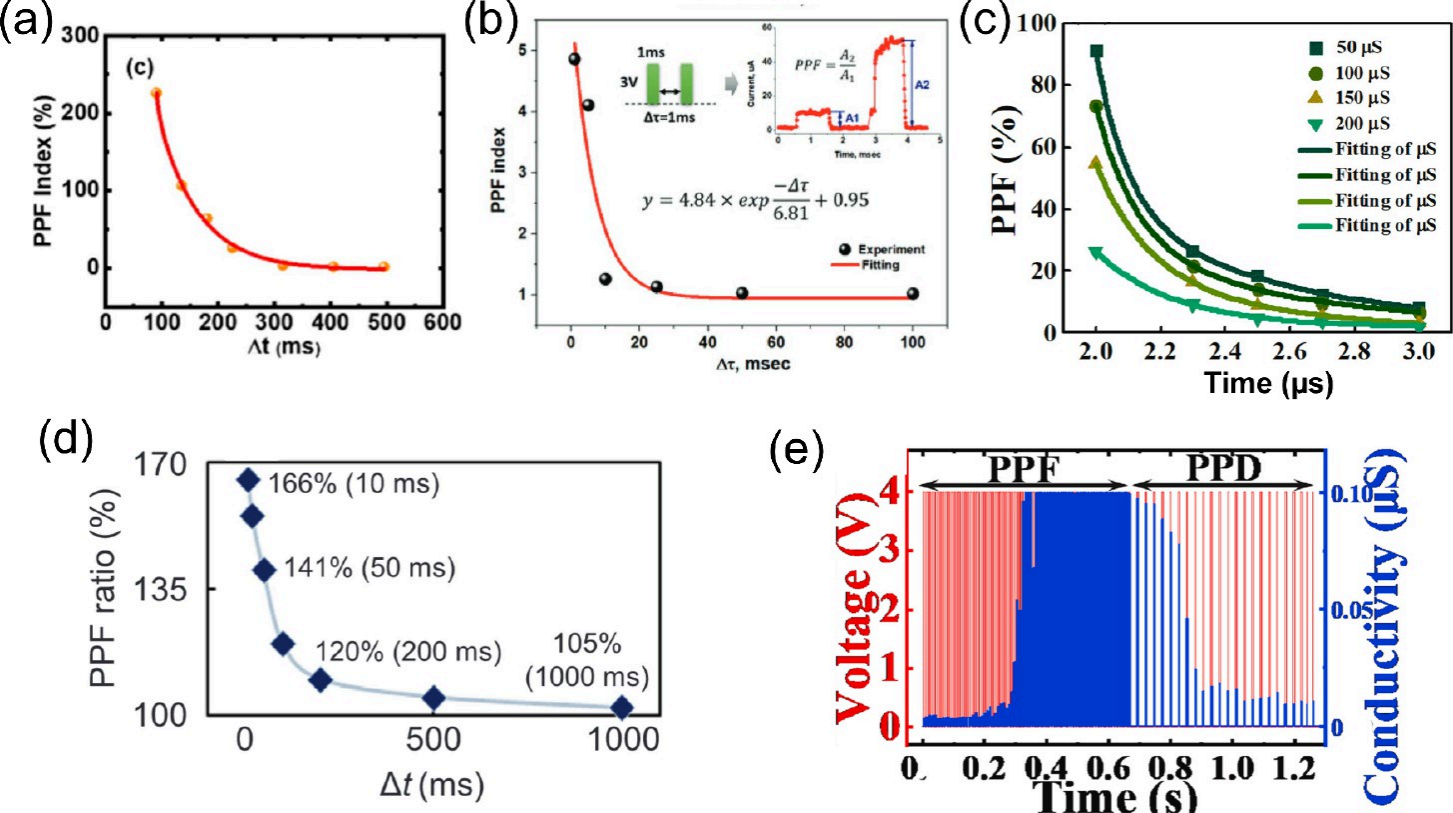
Comparison of PPF index curves in MXene-based devices. (a) Ag/Ti_3_C_2_T_*x*_/SiO_2_/Pt. Reprinted in part with permission under a Creative Commons CC BY License from ref (32). Copyright 2020 Multidisciplinary Digital Publishing Institute. (b) Ag/Ti_3_C_2_T_*x*_-TiO_2_/Pt with partially oxidized Ti_3_C_2_T_*x*_. Reprinted in part with permission from ref (99). Copyright 2021 John Wiley and Sons. (c) Effects of different pulse widths on the PPF index for the Al/Ti_3_C_2_T_*x*_:Ag/Pt stack. Reprinted in part with permission from ref (104). Copyright 2021 Elsevier. (d) Curves for Cu/Ti_3_C_2_T_*x*_/Au. Reprinted in part with permission from ref (98). Copyright 2021 John Wiley and Sons. (e) PPF achievement by modulating the applied pulse frequency in the Ag/V_2_C/W. Reprinted in part with permission from ref (81). Copyright 2021 Elsevier.

As in the human brain, where transient information storage occurs due to the decay to the formed connections, the volatility of RS devices can be used to mimic STP/STD.^141^ For instance, by changing the current compliance (*I*_CC_) in the electroforming of Cu/Ti_3_C_2_T_*x*_/SiO_2_/W, both volatile (*I*_CC_ = 10 μA) and nonvolatile (*I*_CC_ = 500 μA) RS were achieved.^30,31^ Although lower *I*_CC_ values led to higher resistance instabilities, they also resulted in the appearance of neural facilitation associated with STP, as shown by the paired pulse facilitation (PPF) index. Using the same active layer (Ti_3_C_2_T_*x*_/SiO_2_) but with different electrodes (Ag and Pt), Lian et al.^32^ achieved both volatile and nonvolatile behavior. This device showed better PPF values [128 as shown in Figure 9(a) and Table 3], likely due to the lower MXene thickness (2200 to 50 nm) and the replacement of Cu by Ag (lower Gibbs energy). Partially oxidized Ti_3_C_2_ (Ti_3_C_2_T_*x*_/TiO_2_)^99^ showed a high PPF ratio (5× increase) with a 1 ms interval between stimuli (Table 3). This large conductance increase could be attributed to the excessive −F and −O functional groups imparted by thermal oxidation of the MXene. These surface terminations reduce the electrical conductivity, so that the increment in filament density leads to a higher conductance change for stimulus with time intervals smaller than the diffusing relaxation time of Ag^+^. However, the PPF index decays rapidly with rising pulse intervals with no conductance change between consecutive stimulus for pulse intervals >50 ms [Figure 9(b)]. This may be explained by a combination of small pulse width,^142^ together with fast filament decay. The latter is most probably due to the high electronegativity of the Ti_3_C_2_T_*x*_ layer (due to the surface terminations) that increases the Ag^+^ relaxation rate.^99^ In Table 3 we show that this study used one of the shortest pulse widths (1 ms), which should have resulted in a low PPF ratio. The reason for the high value observed can be that the TiO_2_ particles prevent compact stacking of the MXene nanosheets, thus providing defects in the switching layer, which serve as paths for Ag^+^. In these filamentary ECM stacks, there is a trade-off between high PPF values and the operational voltages used. This seems to be influenced by the MXene layer thickness (Table 1), as shown by the low bias on Ag/Ti_3_C_2_T_*x*_/SiO_2_/Pt^32^ (0.2 V, 50 nm) against Ag/Ti_3_C_2_T_*x*_-TiO_2_^99^ (3 V, drop-casting) and Cu/Ti_3_C_2_T_*x*_/SiO_2_/W^30^ (5 V, 2200 nm). In Ag-doped Ti_3_C_2_T_*x*_,^104^ a PPF index of about 95% was achieved at the shortest pulse interval of 2 μs and width of 50 μs. The conductance facilitation decayed rapidly with increased pulse intervals above 3 μs. Interestingly, in this case, increasing the pulse width (up to 200 μs) decreased the PPF index for increasing pulse intervals, as observed in Figure 9(c). In contrast, the lateral Cu/Ti_3_C_2_T_*x*_/Au device^98^ showed PPF on a time scale larger than usual, demonstrating neuron facilitation even with pulse intervals of 1000 ms [Figure 9(d)]. A possible explanation could be the growth direction of the Cu filament (from AE to CE) that creates a reservoir of Cu ions near the Cu electrode, thus reducing the conductance decaying rate. The demonstration of PPF in nonvolatile modes is uncommon, since it is a property inherent to volatile switching. Nevertheless, it was shown that V_2_C^81^ demonstrated PPF in both modes. For the nonvolatile mode (*V* = 4.0 *V*, *I*_CC_ = 10 mA) the device showed low PPF_max_ and a fast decay for the pulse width of 4.0 ms (Table 3). In the threshold (TS) mode (*V* = 4.0 *V*, *I*_CC_ = 0.1 mA, *t*_p_ = 0.5 ms), the device conductance was shown to vary with the applied stimulus frequency, thus demonstrating spiking-rate-dependent plasticity (SRDP). Therefore, obtaining paired-pulse depression (PPD) could be achieved through the modulation of pulse frequency instead of the common polarity inversion [Figure 9(e)]. It was shown that VCM-based Pt/Ti_3_C_2_T_*x*_/Pt stacks^95^ achieved PPF with the index decaying slowly and some neuronal facilitation seen even with pulse intervals reaching 1 s. These results likely stem from the nonvolatile mode together with very long pulse widths (200 ms). Al/Ti_3_C_2_T_*x*_/Pt electronic-based devices with TAT behavior^90^ showed one of the lowest time scales for this neuronal facilitation behavior with MXenes (see Table 3). Such fast decay could arise from the enhanced speed inherent to trap-assisted tunneling in comparison to metal ions. Neuronal facilitation on a large time scale was reported for Au/LPE/Ti_3_C_2_T_*x*_/Si,^91^ also showing extremely low power consumption (460 fW) with high sensitivity of up to 10 mV presynaptic stimulus. Furthermore, it demonstrated tunable synaptic potentiation by modulating the duration, number, and frequency of the applied pulses. Finally, PPF has also been demonstrated in a ferroelectric-based Ti_3_C_2_T_*x*_/PZT device,^63^ showing neuronal facilitation with common decaying time scale and PPF ratios (Table 3). Volatile behavior with PPF characteristics was observed in Ti_3_C_2_T_*x*_/BFO,^62^ using a lower *I*_CC_ value for a regime of only ferroelectric switching.

**Table 3:** STP Performance through PPF of MXene Memristors^a^

Stack	RS Mechanism	*V*_pulse_ (V)	*I*_cc_ (μA)	P. Width (ms)	PPF_max_ (%)	PPF (∼100 ms)	STP to LTP
Al/Ti_3_C_2_T_*x*_/Pt^90^	VCM	4	50	0.01–0.05	∼80	0	Yes
Al/Ti_3_C_2_T_*x*_:Ag/Pt^104^	ECM	2	n.r.	0.05	∼95	0	Yes
Cu/Ti_3_C_2_T_*x*_/SiO_2_/W^30^	ECM	5	10	20	150	2.5	No
Cu/Ti_3_C_2_T_*x*_/PZT/Pt^63^	FE	4	100	20	100	∼37.5	No
Cu/Ti_3_C_2_T_*x*_/BFO/Pt^62^	FE	0.5	10	20	172	∼45.6	Yes
Cu/Ti_3_C_2_T_*x*_/Au (lateral)^98^	ECM	2	n.r.	20	166	130	No
Ag/Ti_3_C_2_T_*x*_-TiO_2_/Pt^99^	ECM	3	50	1	390	0	Yes
Ag/Ti_3_C_2_T_*x*_/SiO_2_/Pt^32^	ECM	0.2	0.1	90	188	128	No
Ag/V_2_C/W^81^	ECM	4	100	4	47	0	No
Au/LPE/Ti_3_C_2_T_*x*_/Si^91^	Li^+^ Diffusion	5	n.r.	n.r.	n.r.	120	Yes
Pt/Ti_3_C_2_T_*x*_/Pt^95^	VCM	4	500	200	∼90	∼55	No

a n.r. stands for non-reported values, and FE stands for ferroelectric. The PPF ratios were calculated from the data points of the figures in the cited works.

#### Long-Term Potentiation and Depression

**Figure 10:**
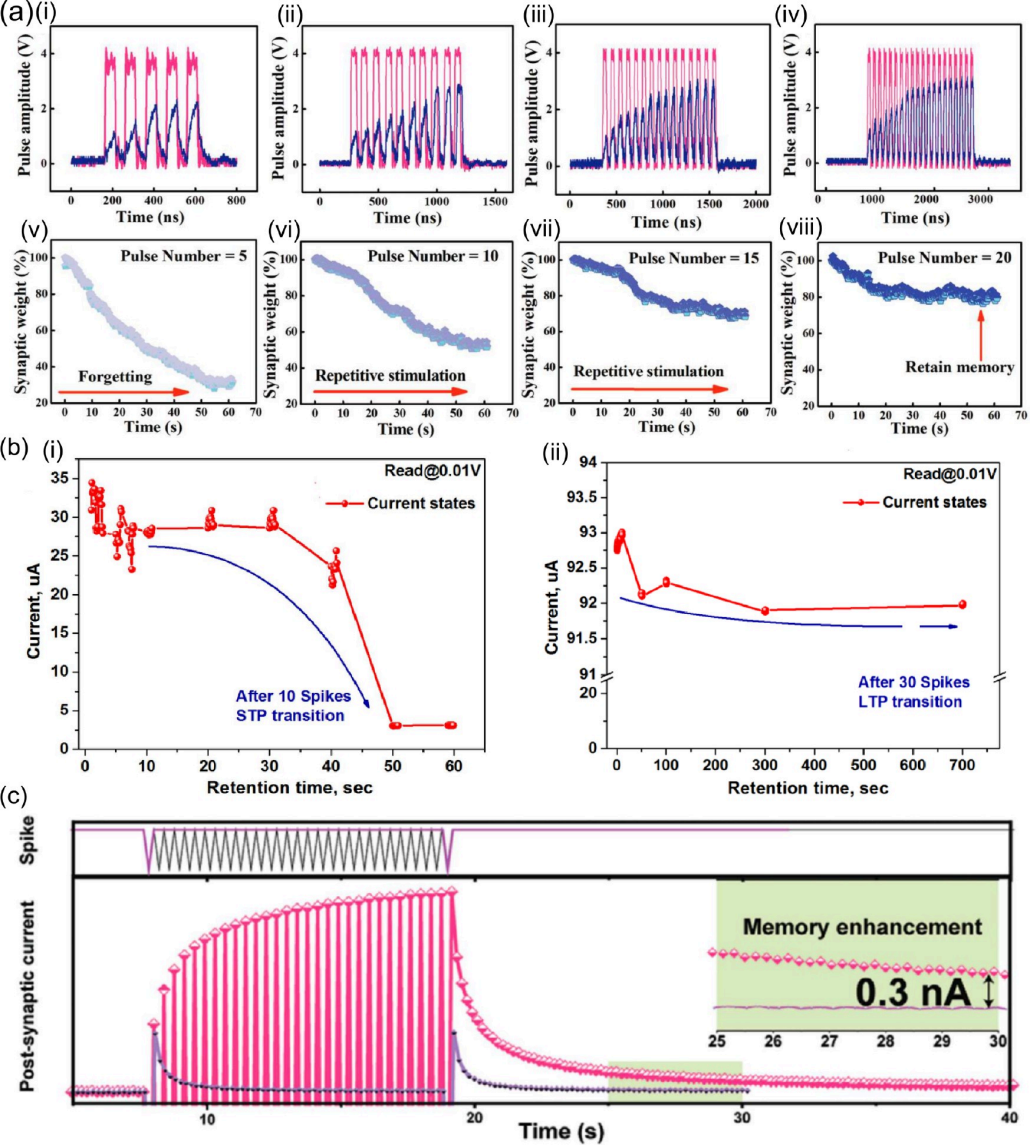
STP–LTP transition in MXene-based devices. (a) Al/Ti_3_C_2_T_*x*_/Pt device transition with increasing pulse number, using nanosecond pulse stimulus (i–iv), resulting in longer retention values (v–viii). Reprinted in part with permission from ref (90). Copyright 2019 John Wiley and Sons. (b) Current after 10 (i) and 30 (ii) pulses on the Ag/Ti_3_C_2_T_*x*_-TiO_2_/Pt. Reprinted in part with permission from ref (99). Copyright 2021 John Wiley and Sons. (c) Memory enhancement in Au/LPE/Ti_3_C_2_T_*x*_/Si after 30 operations compared to 1. Reprinted in part with permission from ref (91). Copyright 2021 John Wiley and Sons.

Through repeated stimulation, STP can be converted to LTP, without changing the applied stimulation characteristics. Al/Ti_3_C_2_T_*x*_/Pt^90^ shows an STP to LTP transition [Figure 10(a.i–a.iv)] by maintaining the same stimulation intensity, duration, and frequency but increasing the number of pulses. It is worth mentioning that pulse intervals and durations (50 ns) significantly lower than the majority of reported studies were used. This transition was also achieved for the partially oxidized Ag/Ti_3_C_2_T_*x*_-TiO_2_/Pt^99^ stack, in the complete volatile regime (*I*_CC_ = 50 μA). In this case, the use of identical parameters as in the PPF experiment was able to maintain the ON state (even after 700 s) by increasing the number of pulses from 10 to 30 [Figure 10(b)]. Instead of tuning filament dynamics, Zhang et al.^62^ leveraged the natural volatility of Cu/BFO/Pt to achieve the STP–LTP transition, by introducing a MXene layer (Cu/Ti_3_C_2_T_*x*_/BFO/Pt). With a low number of pulses, the ferroelectric effect is predominant, but there is a loss of polarization with time (due to the depolarizing field coupled with the low remanent polarization of BFO). However, by increasing the number of stimuli, oxygen vacancies overcome the depolarizing field, thus changing the behavior from STP to LTP. Lastly, an STP–LTP transition was shown in Au/LPE/Ti_3_C_2_T_*x*_/Si with negative differential resistance.^91^ A modulation of the conductance response is seen by increasing the number of applied pulses from 1 to 10, demonstrating spike-duration-dependent plasticity with the conductance rapidly decaying. However, by further increasing the number of applied pulses from 10 to 30, a memory enhancement was observed [Figure 10(c)].

**Figure 11:**
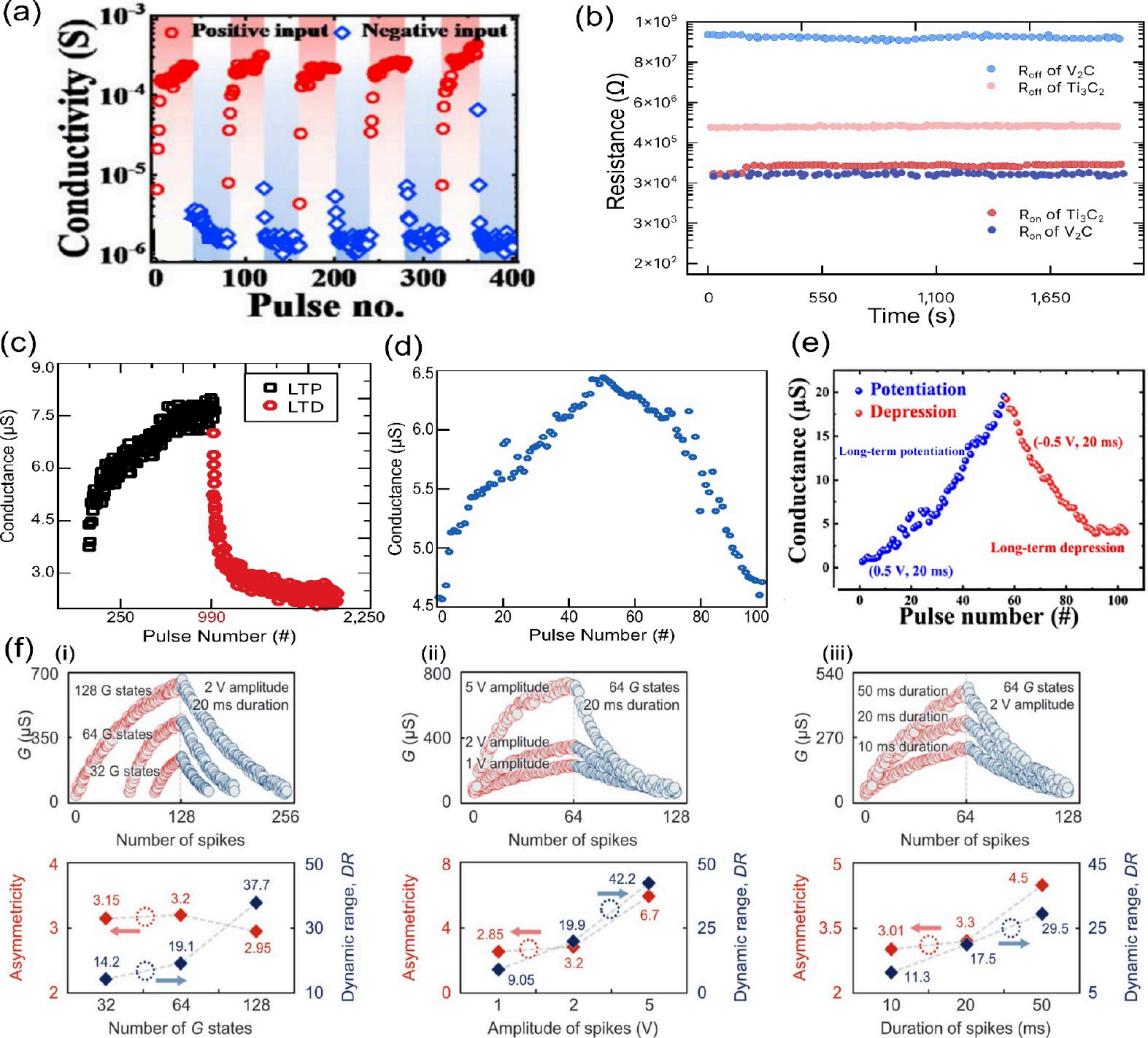
(a) Conductance update of Ag/V_2_C/W, showing a large nonlinearity and asymmetry. Reprinted in part with permission from ref (81). Copyright 2021 Elsevier. (b) Improved retention and endurance of the V_2_C device over the Ti_3_C_2_T_*x*_ one (adapted from values extracted from ref (143)). (c) Demonstration of a large number of levels in the Ag/V_2_C/TiO_2_/W (adapted from values extracted from ref (80)). (d) Symmetric nonlinearity obtained with the Ag/AlOx/Ti_3_C_2_T_*x*_/ITO device (adapted from values extracted from ref (93)). (e) Conductance curves of the Cu/BFO/Ti_3_C_2_T_*x*_/Pt device. Reprinted in part with permission from ref (62). Copyright 2023 Elsevier. (f) Exploration of the effects of pulse stimulus energy and #G levels on the AS and DR. Reprinted in part with permission from ref (98). Copyright 2021 John Wiley and Sons.

Most MXene-based memristors have a gradual current response and can maintain its conductance state long enough to emulate long-term synaptic plasticity, as can be seen in Table 4. Therefore, it is more interesting to analyze which offer enhanced FoMs (Equations 2 to 4) and other relevant parameters and compare them to their learning accuracy (when available). Few MXenes have already been explored for neuromorphic applications besides Ti_3_C_2_T_*x*_. Since different MXenes will have different properties, these can be used to study the performance of memristive devices. The use of V_2_C with Ag as an active electrode (Ag/V_2_C/W^81,143^) offers the largest dynamic range among the reported MXene works and is forming free. However, it has a high NL and AS, as seen in Figure 11(a), which may be due to its asymmetric SET and RESET voltages. This, coupled with its low endurance, would not make it ideal for long-term synaptic applications. Experimental results of Ag/V_2_C/W against Ag/Ti_3_C_2_T_*x*_/W stacks^143^ show that V_2_C improves the performance by lowering both the cycle-to-cycle and device-to-device variations drastically, while increasing the endurance. Furthermore, first-principles simulations demonstrate that V_2_C has an excellent Ag^+^ diffusion coefficient, stabler structure, and enhanced resistance ratio when compared to Ti_3_C_2_T_*x*_ [Figure 11(b)], which explains its very high DR and better C2C and D2D. The same stack was modified to incorporate an additional TiO_2_ layer,^80^ lowering NL, AS, and *I*_CC_ and removing the need of a forming step. Furthermore, it raised #G levels [up to 990 levels, as seen in Figure 11(c)]. Unfortunately, the dynamic range lowered severely to 3.9, which, for such a large number of states, will lead to limited accuracy.^71^

**Table 4:** FoMs of MXene-Based Memristors for Learning Applications^a^

				Pulse												
				Amp. (V)		Width (ms)										
Stack	Switching	*V*_form._ (V)	*I*_CC_ (mA)	P	D	P	D	P. Sym.^i^	# G Levels	DR	AS	NL	Power (nJ)	End. (#)	Ret. (s)	Accuracy^f^ (%)
Al/Ti_3_C_2_T_*x*_/Pt^90^	TAT	Free	50	2	–2	10^–5^	10^–5^	I	20	4.5	7.98	5.4/–2.58^b^	n.r.	10^6^	10^5^	-
Al/Ti_3_C_2_T_*x*_:Ag/Pt^104^	ECM	Free	-	2	–2	5 × 10^–4^	5 × 10^–4^	I	50	2.97	2.55	1.37/–1.18^c^	3.5 × 10^–4^ d	10^6^	10^5^	-
Al/Ti_3_C_2_T_*x*_-TiO_2_ NF/Pt^101^	VCM	Free	-	0.01–0.5	–0.01 to −0.5	5	5	I	50	2.31	0.02	–0.45/–0.47^c^	n.r.	10^4^	10^5^	60–81^h^
Cu/Ti_3_C_2_T_*x*_/SiO_2_/W^30^	ECM	6	500	1.2	–2.2	20	20	N.I.	50	1.19	8.55	3.95/–4.6^b^	n.r.	800	10^3^	-
Cu/Ti_3_C_2_T_*x*_/Au^e^^98^	ECM	3	-	2	–2	20	20	I	128	24.3	3.7	1.49/–1.54^c^	1.42	100	10^4^	93.5
Cu/Ti_3_C_2_T_*x*_/PZT/Pt^63^	FE/ECM	Free	-	1	–1	20	20	I	40	41	6.08	4.26/–1.82^c^	n.r.	100	3 × 10^4^	95.13
Cu/Ti_3_C_2_T_*x*_/BFO/Pt^62^	FE/VCM	Free	-	0.5	–0.5	20	20	I	50	30	0.04	–1.63/–1.67^c^	n.r.	100	10^3^	95.13
Ag/Ti_3_C_2_T_*x*_/SiO_2_/Pt^32^	ECM	1	500	0.2	–0.2	25	25	I	100	2.44	4.9	9.4/–4.43^b^	-	100	10^4^	-
Ag/Ti_3_C_2_T_*x*_-TiO_2_/Pt^99^	ECM	20	250	3	–3	1	1	I	30	-	-	-	18.82	-	-	-
Ag/AlOx/Ti_3_C_2_T_*x*_/ITO^93^	ECM	Free	1000	2	–2	10	10	I	50	6.77	0.41	1.35/1.75^b^	n.r.	100	10^4^	90
Ag/V_2_C/W^81^	ECM	Free	1000	4	–5	8	8	N.I.	40	40	50.5	4.35/–46.14^b^	4 × 10^10^ g	50	4 × 10^3^	-
Ag/V_2_C/TiO_2_/W^80^	ECM	1.7	400	1	–1.2	20	20	N.I.	990	3.9	19.8	2.56/–17.3^b^	2.48 × 10^14^^g^	200	4 × 10^3^	-
Au/LPE/Ti_3_C_2_T_*x*_/Si^91^	Diffusion of Li+	-	-	-	-	-	-	I	50	-	18.9	11.7/–7.2^b^	0.025	-	30	-
Pt/Ti_3_C_2_T_*x*_-TiO_2_/ITO^96^	VCM	Free	-	1	1	10	10	I	100	5	10.36	–8.23/–2.13^c^	n.r.	10^3^	10^4^	-

a n.r. stands for non-reported values and “-” where that parameter does not apply.

b Calculated.

c Reported.

d Per spike.

e Lateral stack.

f For the MNIST data set.

g Only energy specified (nW).

h Image edge detection using the Columbia Object Image Library data set.

i Symmetric or non-symmetric pulses.

As mentioned, filamentary-based devices suffer from fluctuations due to the randomness of the conductive filament formation, making them less reliable for learning applications. Therefore, in addition to the FoMs presented above, there is the need to improve the endurance and stability performances. As discussed for ECM, the introduction of a Ti_3_C_2_T_*x*_ layer in SiO_2_ devices (with Ag or Cu electrodes) lowers the randomness of the CF formation, improving the *V*_SET_ uniformity and providing better stability. This enhanced stability allowed the devices to form stable CFs and achieve long-term potentiation and depression.^30−32^ This behavior was also seen with V_2_C, where it improved the performance of Ag/TiO_2_ devices.^80^ This demonstrates that MXenes are great candidates as functional layers to improve resistive switching reliability (Table 4).

The choice of the top electrode is, also in this case, crucial. Khot et al.^100^ studied these effects of (Ag, Pt, Al)/Ti_3_C_2_T_*x*_/Pt. The Al device showed better endurance and memory retention since it formed an AlO_*x*_ layer during deposition, adding more oxygen vacancies to the MXene layer. MXene doping has also been used as an effective way to decrease device fluctuations. The doping of Ti_3_C_2_T_*x*_ with Ag nanoparticles^104^ enables a forming-free device, a 10-fold energy reduction, and one of the largest endurance values for MXenes. At the same time, it allowed changing RS to an analog behavior. Yan et al.^90^ achieved LTP and LTD with a very low pulse duration of 10 ns, using TAT-based switching with Al/Ti_3_C_2_T_*x*_/Pt. Moreover, this device presented excellent retention and endurance (Table 4) even after a month. Unfortunately, the conductance response is highly asymmetric (Table 4), likely due to the inherent trap-assisted tunneling mechanism, which induces a more abrupt SET and a gradual RESET. Therefore, NL is higher for the potentiation than for the depression.

The linearity can be improved by using additional external circuitry^144^ (although it can be quite complex) or by using algorithms.^145,146^ Nevertheless, the optimal way of improving linearity is by tuning the structure of the stack, such as by the introduction of a buffer layer^147,148^ to prevent abrupt conductance switching, or by controlling the amount of oxygen vacancies.^149,150^ As mentioned, the use of Ag and inherent filamentary RS will result in high nonlinearities (Table 4). Replacing the Ag electrode by Al while doping the MXene layer with Ag greatly enhanced the linearity.^104^ Keeping the Ag electrode but introducing an AlO_*x*_ layer allows positive NL in the RESET process [reversed concavity in the depression part of the conductance in Figure 11(d)]. Such symmetric nonlinearity is rare in analog-resistive switching devices, without any external tuning. This implies close-to-identical changes in the conductance for both potentiation and depression, simplifying convergence efficiency,^71^ while attenuating the effects of asymmetry in degrading learning accuracy.^151^ All of these parameters, together with a close to ideal symmetry (AS = 0.4), explain the simulated 90% accuracy in pattern recognition. As ferroelectric devices tend to be volatile due to the effect of the depolarizing field, they are not suitable for long-term learning applications. Introducing Ti_3_C_2_T_*x*_ in Cu/Ti_3_C_2_T_*x*_/(FE: PZT, BFO)/Pt allowed for a synergetic switching mechanism between the ferroelectric effect and the formation of metal^63^ or oxygen vacancy filaments.^62^ For the case of the PZT/Ti_3_C_2_T_*x*_ stack, the enhanced *R*_ON_/*R*_OFF_ was due to the formation of metallic filaments, which helps to explain the high DR value (Table 4) obtained from the LTP/LTD curves. Unfortunately, the linearity of the potentiation phase is poor (4.26), and it has low #G levels (40), but nevertheless it achieved a high accuracy using the Neurosim+ MNIST test (95.13%). Furthermore, the presence of a metallic Cu filament bridging the PZT/Ti_3_C_2_T_*x*_ layers explains the low linearity in the depression (as it is inherent to filament-based RS). For BFO/Ti_3_C_2_T_*x*_, the *R*_ON_/*R*_OFF_, C2C, and D2D variabilities were also improved. Furthermore, it presents high linearity and symmetry in the conductance curves, as seen in Figure 11(e). This might be due to a combined effect of ferroelectric polarization with oxygen vacancy migration on the modulation of the Schottky barrier formed at the Cu/Ti_3_C_2_T_*x*_-BFO interface, therefore not suffering from the filament dynamics.^1,16,54^ Finally, the authors proved that Ti_3_C_2_T_*x*_/BFO can achieve a very high accuracy in the MNIST simulation (95.13%). Ju et al. used a lateral configuration of Cu/Ti_3_C_2_T_*x*_/Au to leverage the surface terminations on the MXene layer to control the growth of the Cu filament.^98^ The slow growth from the atypical direction of active (Cu) to inert (Au) electrodes most surely contributes to more #G levels with enhanced *ΔG* between them, as reflected in the high DR value (24.3). At the same time, the CF growth on the surface is aided by the surface terminations, which improve the linearity of the conductance change. The device accuracy was explored through online training, revealing that it could achieve a recognition rate of 87.5%, which is very close to the value obtained by an ideal synapse (96.3%). However, its retention and endurance are low, which would make it unsuitable for long-term in-memory learning applications. Pulse parameters were shown to influence linearity, asymmetry, and dynamic range. With equal energy (same pulse width and amplitude) but increasing the number of pulses, the AS value has a small variation, while the DR value increases significantly, as seen in Figure 11(f.i). The #G increase allows higher conductance levels by further growing the filament, thus increasing the DR values. Since CF growth is gradual, increasing the number of identical pulses will have a limited effect in AS. However, if #G continues to rise, AS can suffer because the conductance would start to saturate. With different energies [different pulse amplitude or width; Figure 11(f.i-iii)], both AS and DR increase significantly. Since varying the pulse amplitude or its width is essentially varying the energy amount given to the stack, the growth of the CF will no longer be gradual. Therefore, the larger energy supplied will result in an excess of Cu ions leading to faster filament growth, a more rapid change in conductance, and the deterioration of AS and DR.

#### Spike-Timing-Dependent Plasticity

STDP, one of the key learning mechanisms in the brain based on long-term memory, relates (exponentially) the synaptic weights (*Δw*) with the timing (*Δt*) of pre- and postsynaptic spikes. These learning rules have also been demonstrated in nonvolatile analog memristors by tuning and programming the input pulses’ width, number, and amplitude.^16,152−154^ Although the majority of MXene-based works report neuromorphic long-term plasticity, few demonstrate STDP. In all the successful cases, asymmetric pairs of voltage inputs are used to induce conductance changes, which results in asymmetric STDP, as seen in Table 5.

**Table 5:** Comparison of STDP Properties in MXene-Based Stacks^a^

Stack	Max *Δw* (%)	Pulse Type	Pulse Amp. (|*V*|)
Al/Ti_3_C_2_T_*x*_/Pt^90^	83	Single	4
Ag/V_2_C/Pt^81^	70	Single	4
Cu/Ti_3_C_2_T_*x*_/PZT/Pt^63^	62	Single	4
Cu/Ti_3_C_2_T_*x*_/BFO/Pt^62^	120	Single	0.5

a The behavior observed in published papers is asymmetric Hebbian.

STDP was observed in Al/Ti_3_C_2_T_*x*_/Pt,^90^ with a very low time scale (10 μs), and in Ag/V_2_C/W,^81^ showing that this MXene can also be a good candidate for the emulation of neuromorphic properties. STDP in Ti_3_C_2_T_*x*_/ferroelectrics was achieved by the dominance of filamentary switching over the ferroelectric one: ECM in the PZT/Ti_3_C_2_T_*x*_ stack^63^ and VCM in the BFO/Ti_3_C_2_T_*x*_.^62^

So far, MXenes require high voltages to achieve STDP while having low conductance changes (except for BFO/Ti_3_C_2_T_*x*_; Table 5).^62^

#### In-Memory Learning with MXene Devices

A device which both holds data and performs computing at the same time can not only contribute to reduce power consumption and chip area but also overcome the von Neumann bottleneck.

**Figure 12:**
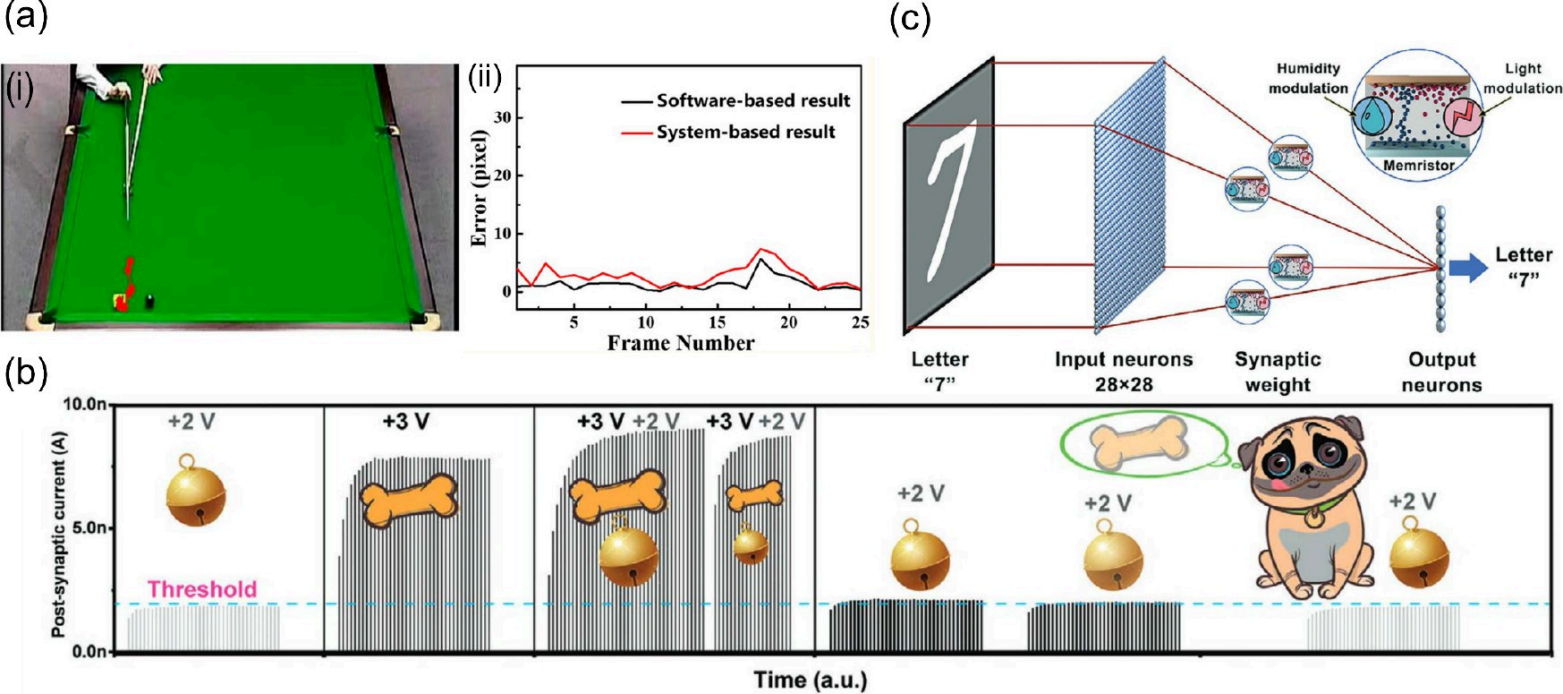
(a.i) Target tracking setup using a memristor; the objective was to track the movement of the white ball. (a.ii) Comparison of the performance of the memristor-enabled algorithm against the software algorithm. Reprinted in part with permission from ref (155). Copyright 2020 American Chemical Society. (b) Demonstration of the famous Pavlov experiment using dual presynaptic inputs in an ionic-MXene device. Reprinted in part with permission from ref (91). Copyright 2021 John Wiley and Sons. (c) Schematics of the setup used for real-time image recognition under different environment conditions. Reprinted in part with permission from ref (103). Copyright 2021 John Wiley and Sons.

Unsupervised learning was achieved in Cu/Ti_3_C_2_T_*x*_/SiO_2_/TiN,^155^ with the successful implementation of parallel multiply and training operations, in particular the implementation of a mean-shift-algorithm-based tracking system [Figure 12(a)].

Even though the Au/LPE/Ti_3_C_2_T_*x*_ stack^91^ did not show clear long-term synaptic capabilities, the accumulation of Li^+^ ions on the MXene layer under an applied voltage enabled tunable synaptic weights, by amplifying consecutive pulses. Additionally, it could respond to presynaptic currents with great sensitivity, with amplitudes similar to those of biological synapses (80 mV) at an astonishingly low power consumption of just 460 fW, most probably due to the EDL created at the interface of the LPE/Ti_3_C_2_T_*x*_. Furthermore, dendritic integration was assessed by using the device with two inputs and one output, where different presynaptic currents were applied to the input terminals and logic operations were successfully performed. The same configuration was used to replicate Pavlov’s experiment where, by continuous training sessions, one stimulus becomes associated with the other input and the output is fired by either of the inputs [Figure 12(b)].^156^

Taking advantage of the electric, optic, and humidity sensing capabilities of the Ti_3_C_2_T_*x*_/ZnO stack,^103^ Wang et al. achieved image recognition that mimics the conditions of the human eye. First, the device is used to preprocess raw data from input images (emulating retinal function), which was then fed to a software-based artificial neural network (ANN). Allowing the ANN to be trained under different environment conditions (relative humidity and light) showed that image recognition under a relative humidity of 40–60% had a better performance than that under 0–20% relative humidity (82.96% and 75.44%, respectively). Furthermore, they used a first layer array (28 × 28) with these artificial neurons, implementing a preprocessing of visual data (functioning as an in-sensor computing unit). It was then connected to 10 output neurons (same composition), which received the current from the previous layer, and then image recognition tasks were performed [Figure 12(c)].

In-memory computing can also be applied for arithmetic calculations, such as the addition and multiplication performed by the Ag-doped Ti_3_C_2_T_*x*_,^104^ with great accuracy, due to its good linearity. In the case of the addition, the current change was calculated at each SET operation. It was shown that, after 20 consecutive stimuli, the device reached a conductance change value of 3.2. Therefore, each time the current percentage reached this value, the number 20 was added to the next operation. Cumulative addition and multiplication calculations were also demonstrated.

## Critical Assessment

There is a rising ubiquity of machine learning and artificial intelligence solutions in a broad range of fields, even already widely accessible through user-friendly interfaces. Such advances come with the great cost of being computationally intensive, leading to a high power consumption. Therefore, there is an urgent need for alternative computing architectures which are efficient but scalable. An emergent solution for this dilemma is 2D memristive devices that demonstrate neuromorphic properties. Ongoing research shows that the substitution of thick materials in the switching layer, for two-dimensional ones, offers lower power consumption and better neuromorphic properties and scalability. Thus, the exploration of the integration of 2D materials in resistive switching devices has exploded in recent years, with a high emphasis on TMDs. Since 2019, research on MXene-based memristors has begun due to their excellent charge trapping capability and electrical conductivity, with growing evidence of their contribution to enhancing neuromorphic properties.

Here, we provided an extensive overview of MXene-based artificial synapses in terms of their fabrication and characterization methods at the material and device level, switching mechanism analysis, and neuromorphic properties.

We gave an in-depth review of the fabrication methods (synthesis and deposition) of the MXene layer, focusing on its application for artificial synapses. The different etching routes for Ti_3_C_2_T_*x*_ were presented, exploring their main advantages and disadvantages, followed by a thorough description of a common protocol for the complete synthesis process. Moreover, a review of the etching process for the less common (in neuromorphic applications) V_2_C was also given. Modifications of the synthesis process, to impart oxidation, defects, and different surface terminations, were presented. The deposition of the MXene layer was explored, mainly by the spin-coating technique due to its prevalence in the reported studies.

**Figure 13:**
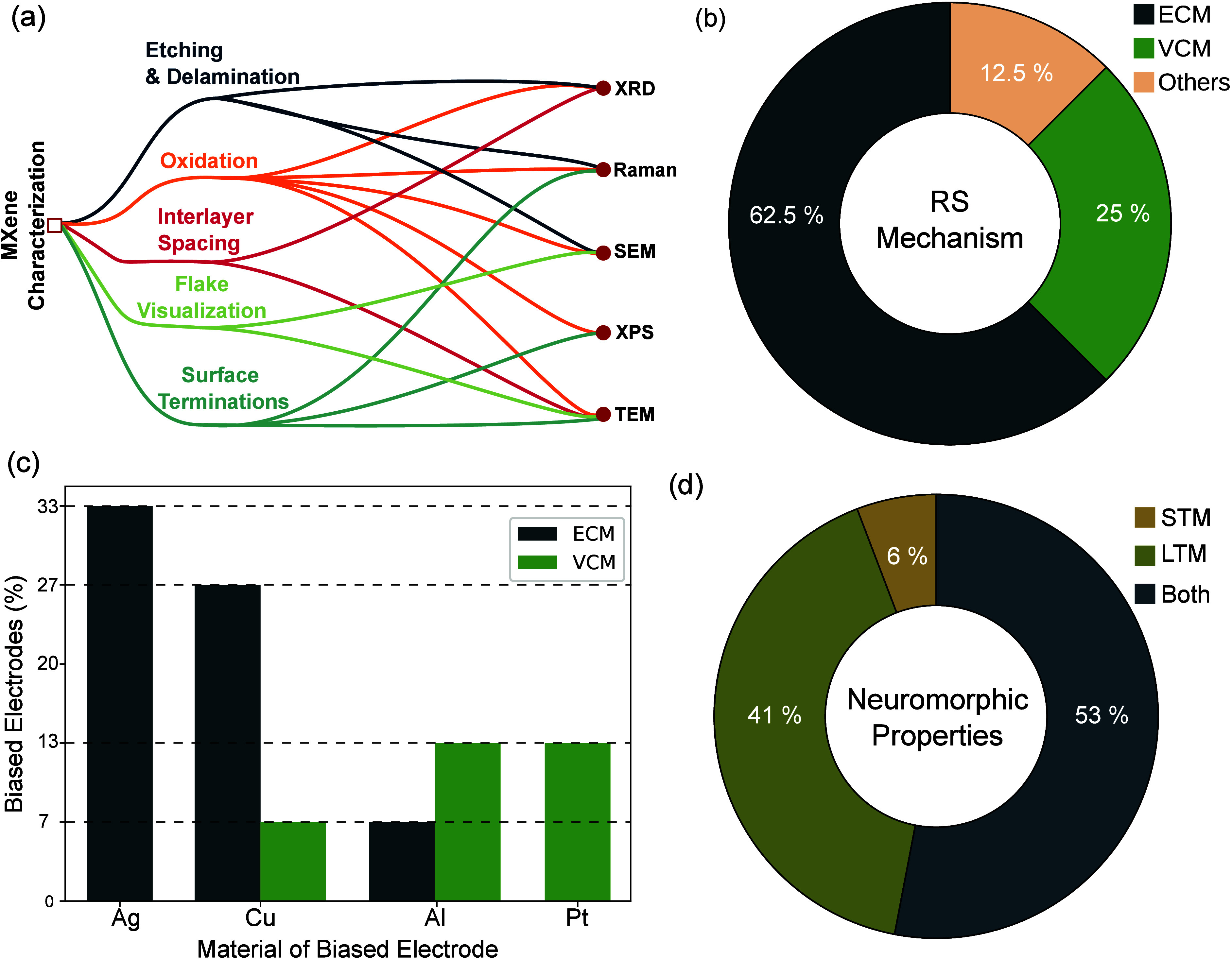
(a) Summary of the most appropriate characterization techniques for each fabrication step: XRD,^40,84,96,123,143^ Raman,^40,96,98,99,117,123,125,157^ SEM,^40,84,101,110^ XPS,^40,90,95,98,101,117^ and TEM.^40,84,90,98,104,116,117^ (b) Percentages of switching mechanisms present in MXene-based memristors for artificial synapses. (c) Representation of the choice of electrodes in the works reviewed here. (d) Distribution of volatile (STM),^99^ nonvolatile (LTM),^31,80,81,93,96,98,101^ or joint^30,32,62,63,90,91,95,98,104^ behavior in neuromorphic properties of MXene-based artificial synapses.

Since MXene properties vary at each step of the fabrication process, the best characterization techniques for each step of the synthesis and deposition were enumerated and are summarized in Figure 13(a).

The switching mechanisms present in MXene-based devices that mimic artificial synapses are dominated by a filamentary ECM [Figure 13(b)]. This is expected since Ti_3_C_2_T_*x*_ possesses inherent Ti vacancies that act as acceptors. Therefore, with the majority of works choosing active electrodes [Cu and Ag; Figure 13(c)], the distributed vacancy sites attract the dissolved cations from the active electrode that drift into the MXene layer with the applied bias. The resulting decrease in the randomness of filament formation leads to increased device stability (better state definition, higher endurance and retention, and better C2C and D2D) and a decrease in operating voltages, as seen in the devices with Ti_3_C_2_T_*x*_/SiO_2_ heterostructures.^30−32,94,129,133^ However, the reactive nature of stacks with Ag or Cu electrodes will degrade the performance over time. To prevent this, doping of Ti_3_C_2_T_*x*_ with Ag^+^ leverages (again) the use of Ti vacancies, enabling the trapping/detrapping of Ag^+^,^104^ forming filaments without electrode degradation. Furthermore, MXene surface terminations also play a role in attracting positively charged species due to their electronegativity. This was cleverly employed in the Cu/Ti_3_C_2_T_*x*_/Au lateral stack,^98^ where the growth of Cu filaments is mediated by the existing surface terminations which slow down their growth, changing it from AE to CE [Figure 7(f)]. Finally, to tackle the good conductivity of MXenes (which leads to small *R*_ON_/*R*_OFF_ in memristors with MXenes as a single resistive layer), oxidation of Ti_3_C_2_T_*x*_ can also be performed.^99^

From Figure 13(c), we see that a small number of works have chosen inactive metals for both electrodes. In these, VCM is probably the dominant RS mechanism, although some oxidation must be present in the MXene layer to enhance the growth of vacancy-based CFs. This oxidation can be ambient^63,100^ or induced.^96,101^ Both Khot et al.^100^ and Zhang et al.^63^ achieved VCM through ambient oxidation of their MXene layer. However, there is a better performance reported for Al/Ti_3_C_2_T_*x*_/Pt^100^ in comparison with Pt for electrodes in the same study and in ref (63). Although both present the same level of ambient oxidation (through XPS measurements), the deposition of Al forms a thin layer (∼5–10 nm) of AlO_*x*_, that increases the quantity of oxygen vacancies available in the switching layer, improving the filament stability.^100^ Exploration of actively oxidizing Ti_3_C_2_T_*x*_ used annealing (65 °C) to form a TiO_2_ layer,^96^ or an alkalization process to form a porous nanostructure of oxidized Ti_3_C_2_T_*x*_.^101^ Both increase the oxygen vacancies present in Ti_3_C_2_T_*x*_ but at the same time increase the number of surface terminations, which also aid in vacancy diffusion. Electrodes that enhance the vacancy reservoir, either ITO^96^ or Al (to form AlO_*x*_^101^), were also used. This shows that MXene-based devices showing VCM behavior, even though scarce, can be improved by the choice of electrodes, the oxidation state of the MXene layer, or the introduction of an oxide layer (as heterostructures or through Ti_3_C_2_T_*x*_ oxidation).

Heterostructures of ferroelectrics with MXenes (Ti_3_C_2_T_*x*_) have started to be explored, where the addition of the MXene layer lowers the energy barrier at the interface for easier charge crossing, allowing the bridging of a CF.^62,63^ One work^62^ shows the coexistence of volatile and nonvolatile regimes, by employing different *I*_CC_, that in turn control whether the dominant mechanism is ferroelectric or VCM. Nevertheless, due to the complicated nature of ferroelectric switching in heterostructures, more in depth studies should be performed, including STEM- or temperature-dependent measurements of devices in the ON and OFF states to understand the nature of the filament formed.

Forming is present in the majority of devices that show filamentary ECM and have an oxide layer in the stack (SiO_2_,^30−32^ TiO_2_^80,99^), because they need a higher energy to introduce ions to the dielectric. In contrast, works that use thin MXene layers,^81^ or the doping of these with conductive nanoparticles,^104^ work with VCM behavior by oxidizing the MXene and choosing appropriate electrodes^96,101^ or on leveraging the MXene/ferroelectric heterostructure, do not show the need of a forming step.^62,63^

The works reviewed present neuromorphic properties by emulating synapses either in short- (volatile) and/or long-term (nonvolatile) learning process, as presented in Figure 13(d). One of the main indicators for good short-term synaptic learning is PPF performance. From Table 3, the devices with better initial PPF ratio are filamentary^32,98,99^ and ferroelectric.^62^ From the filamentary ones, the device with the highest PPF is Ag/Ti_3_C_2_T_*x*_-TiO_2_/Pt,^99^ due to the increase in initial resistivity coupled with fast filament formation, aided by surface terminations. Nevertheless, some with high PPF ratio needed higher operational voltages due to a thicker MXene layer (Table 1). Lateral Cu/Ti_3_C_2_T_*x*_/Au shows a high PFF ratio due to the surface terminations for the Cu cations to drift.^98^ Lastly, Cu/Ti_3_C_2_T_*x*_/BFO/Pt^62^ demonstrates good PFF with low voltage, by tuning *I*_CC_ to set the device to the ferroelectric regime only.

To use MXene-based devices in learning applications, they have to meet strict requirements in performance level, which are dictated by FoMs. From the works reviewed here, those with the best endurance are forming free (Ag doping of Ti_3_C_2_T_*x*_^104^) or exhibit a purely electronic mechanism (TAT^90^), preventing the need of filament formation or chemical changes. These also use the lowest pulse width due to their switching mechanism. The dynamic range should be above 10, for great accuracy in learning applications, but few works have achieved that target. As a single layer, the Ag/V_2_C/W stack^81^ and the lateral Cu/Ti_3_C_2_T_*x*_/Au device^98^ comply with this parameter. The heterostructures with ferroelectric layers^62,63^ both achieve larger DR values, most probably due to the dielectric nature of the ferroelectrics versus the metallic nature of the filaments. Interestingly, the works using oxidation of Ti_3_C_2_T_*x*_,^96,101^ to increase the oxygen reservoir (thus not needing a forming step), suffer from the higher conductivity in the OFF state. Therefore, their *R*_ON_/*R*_OFF_ values are not very high. Note that, as seen by the works of Ju et al.^98^ [Figure 11(e)], FoMs can be tuned to optimal values or greatly deviate from these in the same device, demonstrating the paramount importance of tuning the pulse parameters used.

Using these devices to realize the brain’s complex computations through hardware implementation of artificial synapses will offer an alternative to von Neumann chips. Most of the works reviewed display good benchmark values for digit recognition tasks. However, they are simulated using the Neurosim+^158^ algorithm, and therefore no real application was so far employed. Some MXene-based memristors have already been used for in-memory computing in tasks such as visual tracking systems,^155^ dendritic integration and logic operation,^91^ image recognition sensitive to humidity and light,^103^ and arithmetic calculations.^104^

## Outlooks

Although MXenes were discovered only a decade ago, they are quickly being adopted into a broad range of research areas, such as neuromorphic computing. We are still in the early ages of MXene-based neuromorphic devices, and already challenges are posed such as device reproducibility, stability, or its use as single-layer resistive switching. At the same, there is an opportunity for the systematic exploration of the vast MXene family and the extensive tuning of its properties.

### Challenges

#### Synthesis and Assembly

Even though a lot of research has been put into MXene synthesis for a vast array of applications, it is certain that its process can confer very different properties to the finished product by introducing variability in the defects, oxidation, flake size, surface terminations, interlayer spacing, and more. Although having a large number of variables that can be changed during the synthesis is extremely interesting for tuning MXene’s properties, it poses a problem for device reproducibility. At the same time, the deposition process used (even though it is cheap and practical) is prone to introduce incomplete surface coverage and variability, also deeply affecting the resistive switching mechanism. Thus, improvements in MXene synthesis uniformity and deposition must be addressed for both small- and large-scale production of devices.

It has already been seen that organic solvents and solution concentration optimizations help in this regard. However, vacuum or freeze-drying of MXenes to then redisperse them in the solvent of choice can be very difficult, especially for the LiF/HCl etching, such that intensive sonication is required. This will result in reduced flake size but also induce oxidation and defects. One solution can be to use a solvent exchange method, which has proven to work for a vast array of organic solvents without damaging or reducing the size of the flakes.^123^

For industrial production there is a strict need to control the film’s uniformity and thickness. This could be achieved by developing atomic layered deposition or chemical vapor deposition. Nevertheless, the layer-by-layer method^121,159^ (already used in MXene neuromorphic devices^92^) is also an excellent low-cost candidate to meet these needs.

#### Tuning of MXene layer

Modifying the MXene’s flakes is commonly performed in other areas, to fine-tune their physicochemical characteristics and obtain enhanced results. The same procedure is starting to be performed for MXene-based memristors, including by doping with nanoparticles^104^ or induced oxidation.^96,99,101^ Although these modifications enabled better performance (e.g., increased retention and endurance) and forming-free, they still suffer from low *R*_OFF_/*R*_ON_ (Table 4). The mentioned studies use modified Ti_3_C_2_T_*x*_ as a single layer and obtain excellent results in terms of stability; however, the same modifications are not enough to tackle the high conductivity of MXenes (even with oxidation), inhibiting good neuromorphic capabilities. Therefore, using MXenes as the sole RS layer for neuromorphic applications must take into account their high conductivity but ensure that the tuning is more holistic.

#### Stability

For long-term learning applications, the fabricated devices should not suffer significant degradation. The other major culprit in device degradation is oxidation of the MXene sheets. Therefore, addressing their environmental stability should be prioritized (e.g., by edge-capping or functionalization through conjugated polymers). Since the majority of reports are based on filamentary switching, it is expected that degradation will occur due to the chemical changes innate to the switching mechanism when updating the state of the device. Therefore, solutions based on other RS mechanisms, such as ferroelectric switching, should be sought. For example, various MXenes (including Ti_3_C_2_T_*x*_) can be made ferroelectric with facile synthesis steps.^160^

#### Neuromorphic Performance

MXene-based memristors can emulate the basic functions of biological synapses. However, they are still behind in some key FoMs necessary to realize complex functions. The first issue to address is the endurance, which is especially affected by filamentary switching. Strategies to eliminate the forming process should be sought, since it naturally changes the structure of the switching layer, leading to lower resistance and endurance, higher fluctuations, and lower *R*_ON_/*R*_OFF_. Note that some MXene-based devices already demonstrated forming-free switching, as seen in Table 4.^81,93,104^ Second, the linearity has to be improved, as well as the symmetry that is greatly affected by the dynamics of filamentary switching. Finally, a more holistic solution of emulating biological synapses has to encompass the fabrication of devices with in-memory computing, incorporating sensors.^104^

### Future Directions

MXene research has grown every year since 2014, in the number of applications, properties, or materials. The same applies to the research on MXene-based devices for neuromorphic computing. Some possible future research directions are suggested.

#### Other MXenes

The MXene family is very vast, with MXenes being predicted and synthesized at a fast pace. However, studies for neuromorphic applications are Ti_3_C_2_T_*x*_-centered. Other MXenes with similar synthesis processes as Ti_3_C_2_T_*x*_ should be studied to try to achieve better neuromorphic properties. From the few examples with V_2_C we see that, although not with the best overall performances, they offer the best DR and #G values. At the same time, V_2_C shows better oxidation stability than Ti_3_C_2_T_*x*_, enduring up to 350 °C. Modification of well-known Ti_3_C_2_T_*x*_ through the use of heat-treatment has employed ferroelectric properties, which have been used as a switching layer to obtain nonvolatile digital RS devices.^160−162^ Therefore, these modifications can be advantageous to obtain resistive switching without relying on filament formation and without resorting to stacking other materials. Furthermore, other MXenes could also be explored since various theoretical studies support the existence of ferroelectric phases in different MXenes^163−165^ and there are digital memristors which employ this tuning.^161^ For the case of Li^+^ intercalation resistive switching devices,^91^ theoretical work has shown that M_2_X MXenes have stronger bonds between M and X than M_3_X_2_, which means an easier diffusion of ions between the MXene sheets of M_2_X-type.

#### Relationship between MXene Properties and Resistive Switching

The impact of MXene’s electrical properties such as energy bands or work function is still not properly investigated in the context of neuromorphic applications. Although there is vast literature on the tuning of these properties for other applications (both experimental and/or theoretical), for neuromorphic applications only the matching of the work function has been explored.^62^ At the same time, surface terminations also affect these properties. Even more importantly, surface terminations impact the stacking with other materials, play a role in the oxidation of MXenes, and impart different electrical conductivities and structural properties. Therefore, it is of paramount importance to investigate the relationship between surface terminations and the performance of the memristors.

Similar to surface terminations, the interlayer spacing is a parameter that can be easily tuned during the synthesis process and greatly affects the capacitance of MXenes by controlling the size of the intercalating ions. The intercalation of Ti_3_C_2_T_*x*_ with Li^+^ enabled neuromorphic behavior.^91^ Moreover, it has been shown that −Cl terminated Ti_3_C_2_ offers the lowest diffusion barrier for Li^+^.^166^ Therefore, ion intercalation devices might be an interesting way to prevent structural degradation on ECM/VCM.

Lastly, the resistive switching dynamics should be corroborated by *in situ* TEM/AFM studies, as proved by the observation of defect evolution in other 2D materials through *in situ* HRTEM,^167,168^ which has not yet been explored in MXene-based memristors.

#### Heterostructures

MXenes have great compatibility with other 2D materials due to their van der Waals interactions, allowing vertical layer-by-layer two-dimensional heterostructures. These heterostructures retain their individual properties, and therefore are a great opportunity to explore 2D materials commonly used in neuromorphic applications with MXenes. Moreover, it has been seen with Ti_3_C_2_T_*x*_/SiO_2_ stacks that the energy barrier for cations is lowered. This was also observed in digital memristors (Ti_3_C_2_T_*x*_/GST:Ge_2_Sb_2_Te_5_).^169^

Exploration of heterostructures with TMDs is not reported in neuromorphic applications, even though they have great compatibility, with excellent lattice match, and there is vast literature on TMD applications in neuromorphic devices. Furthermore, it may be beneficial to use a modified drop-casting method, as explored in ref (170), since it offers easy thin-film Ti_3_C_2_T_*x*_ deposition with high surface coverage in high dielectric substrates such as MoS_2_ or HfO_2_. Heterostructures with ferroelectric materials should also be explored, since the few examples available presented good results. Furthermore, ferroelectric switching generally has better endurance (since no chemical change is occurring) and higher switching speeds than ionic devices.^16,171^

## Vocabulary

**Biological Learning Mechanisms:**: Refers to the cognitive processes observed in living organisms that enable them to learn and adapt to their environment, through pattern recognition, memory formation, and decision-making. These plasticity mechanisms can be categorized according with the type of change (potentiation/depression), duration (short/long-time), or stimuli timing (time-dependent plasticity; paired-pulse facilitation).
**Memristor:**: A nonlinear passive component defined by a pinched current–voltage hysteresis loop. This two-terminal device remembers its resistance based on the history of the electric current that has flowed through it and can mimic a biological synapse.
**MXene:**: A recent class of two-dimensional (2D) transition metal carbides, nitrides, and carbonitrides. They are characterized by their unique layered structure, high electrical conductivity, and tunable surface chemistry, making them promising materials for a wide range of applications, including energy storage, electronics, catalysis, and neuromorphic computing.
**Neuromorphic Computing:**: Seeks inspiration from the brain to store and process information in the same substrate through networks of artificial neurons and synapses.
**Resistive Switching Mechanisms:**: Explain the change (gradual or abrupt) in the resistance of memristors, under an applied external bias. The mechanisms vary (e.g., ionic, electronic, ferroelectric) depending on the choice of the electrodes and active material.
